# Spatiotemporal single-cell architecture of gene expression in the *Caenorhabditis elegans* germ cells

**DOI:** 10.1038/s41421-025-00790-4

**Published:** 2025-03-18

**Authors:** Lili Li, Xiaoyin Tang, Xuanxuan Guo, Di Rao, Lin Zeng, Junchao Xue, Shuxian Liu, Shikui Tu, En-Zhi Shen

**Affiliations:** 1 College of Life Sciences, Zhejiang University, Hangzhou, Zhejiang China; 2 Key Laboratory of Growth Regulation and Translational Research of Zhejiang Province, School of Life Sciences, Westlake University, Hangzhou, Zhejiang China; 3 Westlake Laboratory of Life Sciences and Biomedicine, Hangzhou, Zhejiang China; 4 Institute of Biology, Westlake Institute for Advanced Study, Hangzhou, Zhejiang China; 5 Department of Computer Science and Engineering, Center for Cognitive Machines and Computational Health (CMaCH), Shanghai Jiao Tong University, Shanghai, China

**Keywords:** Reprogramming, Transdifferentiation

## Abstract

Spermatogenesis is an intricate and tightly controlled process encompassing various layers of gene expression regulation. Despite the advance of our current understanding, the developmental trajectory and regulatory mechanisms dictating spermatogenesis remain elusive. In this study, we have generated single-cell gene expression profiles for *Caenorhabditis elegans* sperm cells and constructed gene regulatory networks alongside the developmental trajectories of these cells. Our findings indicate that each pre- and post-developmental stage is closely linked by co-expressed genes, while simultaneously being uniquely identified by the combined expression of specific gene families. To illustrate the applicability of this exhaustive gene expression catalog, we used gene regulatory networks to uncover potential transcription factors for (1) the expression of genes in the phosphorylation pathway, identifying NHR-23-to-phosphatase regulation for the meiotic cell division process; and (2) the expression of constituent components of small RNA pathways, identifying ELT-1-to-Argonaute protein regulation for siRNA maintenance and sperm activation. We expect that this sperm cell-specific gene expression directory will prompt investigations into the underlying mechanisms determining anatomy, differentiation, and function across the reproductive system. Finally, our expression data can be explored using the web application CelegansGermAtlas (https://scgerm-atlas.sjtu.edu.cn/website/#/home).

## Introduction

Completing genome sequencing has provided us with an exhaustive catalog of genes, crucial to orchestrating and regulating cellular, developmental, and behavioral processes in organisms^1–4^. A critical next step is to understand how transcription factors (TFs) orchestrate the dynamic transcription of these genes in a spatiotemporal manner to drive specific cellular functions.

Germ cells play a pioneering role in ensuring species continuity^5,6^. These cells undergo meiosis to create mature gametes, which, following fertilization, develop into fully formed individuals, thereby facilitating both the preservation and propagation of species. This necessitates germ cells to undertake a continuous process of cell differentiation through meiosis before reaching maturity^6–8^. Male germ cells, in particular, experience spermatogenesis — a multifaceted process involving spermatocytogenesis and meiosis. Spermatocytogenesis includes mitotic divisions to produce spermatogonia and primary spermatocytes, while meiosis ensures genetic recombination and the formation of haploid round spermatids. Given the dynamics of spermatogenesis, our understanding of the spatiotemporal gene expression and their transcriptional regulation within this process remains quite limited.

Over the past several decades, *Caenorhabditis elegans* has risen as a valuable model organism for investigating spermatogenesis, owing to its transparent body, the plenitude of germ cells comprising approximately two-thirds of the total cell population, and an evolutionarily conserved spermatogenesis process^9^. This process initiates with germline stem cells located at the distal region (basement membrane of seminiferous tubule) and concludes with maturing spermatids at the proximal region (lumen). Moreover, *C. elegans* is a self-fertilizing hermaphrodite^10^, and germline development entails the sequential differentiation of male and female germ cells, which eventually mature into sperm and oocytes, respectively. During the fourth larval stage (L4), germ cells mature into spermatocytes and subsequently differentiate into round spermatids. Post late L4, germ cells commence differentiating into oocytes. This model offers a robust platform for exploring fundamental biological processes such as sperm stem cell differentiation and meiotic cell division^11,12^.

Prior studies have suggested a gradual decrease in gene transcriptional activity during germ cell differentiation, implying that spermatocyte differentiation may increasingly rely on post-transcriptional and post-translational activity in the late stage of spermatogenesis to ensure the successful progression of spermatocyte differentiation into spermatozoa^13–15^. MicroRNAs (miRNAs), small interfering RNAs (siRNAs), and PIWI-interacting RNAs (piRNAs) were three major classes of small RNAs^16–18^. Following the breakthrough discovery that small RNA-based pathways emerge as regulatory mechanisms for post-transcriptional control of gene expression in multicellular eukaryotes^19,20^, these RNA species have gained significant attention. Small RNA or DNA guides direct Argonaute proteins to find and bind specific DNA or RNA target sequences, playing crucial roles in developmental timing, tissue differentiation, and protecting the genome integrity from transposon and foreign nucleic acids such as viruses^21–23^. Despite the fundamental biological functions, the expression landscape of components in these different small RNA pathways remains to be explored. Among various post-translational modifications (PTMs), phosphorylation and dephosphorylation have emerged as crucial regulatory mechanisms^13,24–27^. The advancement of spermatogenesis is facilitated by the spatiotemporal-specific expression of kinases and phosphatases. However, their transcriptional regulation and molecular functions during spermiogenesis are not well understood.

Previous studies on gene expression in the germline of *C. elegans* primarily involved isolating gonads, dissecting gonadal sections, or extracting germ cell nuclei^28–30^. With the development of single-cell sequencing technology, Cao et al. isolated cells from the L2 stage, including germ cells, and performed single-cell sequencing. However, germ cells at the L2 stage are only beginning to proliferate, and thus the data do not encompass the complete developmental process of germ cells^31^. Subsequent studies utilized single-cell RNA sequencing (scRNA-seq) to investigate neuronal development and aging in adult *C. elegans*^32,33^; however, these studies did not explore sperm development. As in previous studies^31–33^ using the same method^34^ for cell isolation, we obtained cell suspensions from the L4 stage, when most sperm development occurs. In this study, we focused on germ cells in L4 *C. elegans*, using high-precision scRNA-seq to obtain the transcriptome data and characterize their developmental transcriptome atlas. In addition, we identified cell type-specific transcriptomic signatures and co-expression regulatory networks. To showcase the utility of this extensive gene expression catalog, we utilized gene regulatory networks to discover potential TFs involved in the regulation of the phosphorylation pathway as well as the expression of integral elements of small RNA pathways. We found that the TF NHR-23 regulates the phosphatases, such as W03F11.4, to influence spindle progression from metaphase to anaphase II and the clearance of residual bodies, thereby ensuring male fertility. Another TF, ELT-1, was found to regulate the expression of two Argonautes, ALG-3/4, which are required for the maintenance of endogenous siRNA 26G RNA and the activation of spermatids. Finally, we provided a user-friendly web tool that maps the temporal and spatial activity of different genes in germ cells, providing a useful resource for researchers to study germ cell differentiation and reproduction.

## Results

### Single-cell transcriptomes of the *C. elegans* germ cells

To obtain *C. elegans* germ cells, we utilized CRISPR/Cas9-generated knock-in fluorescent strain, *gfp::tev::flag::prg-1*, which specifically expresses GFP::TEV::FLAG::PRG-1 in germ cells from the endogenous *prg-1* locus. This strain exhibits characteristics of progeny number and lifespan akin to the wild-type (WT) (Supplementary Fig. S1a, b), and we thus used this strain to prepare a single-cell suspension. After synchronization, most animals were at the middle L4 larval stage, with a few at the early and late L4 larval stages (Supplementary Fig. S1c). After collecting the cell suspension, we used density gradient centrifugation to enrich the germ cells (Supplementary Fig. S2a, b) and then isolated a single, viable germ cell using fluorescence-activated cell sorting (FACS) (Supplementary Fig. S2c, d). We generated 13,949 single-cell transcriptomes with an average of 16,395 unique molecular identifiers (UMIs) and 3303 genes per cell.

**Fig. 1:**
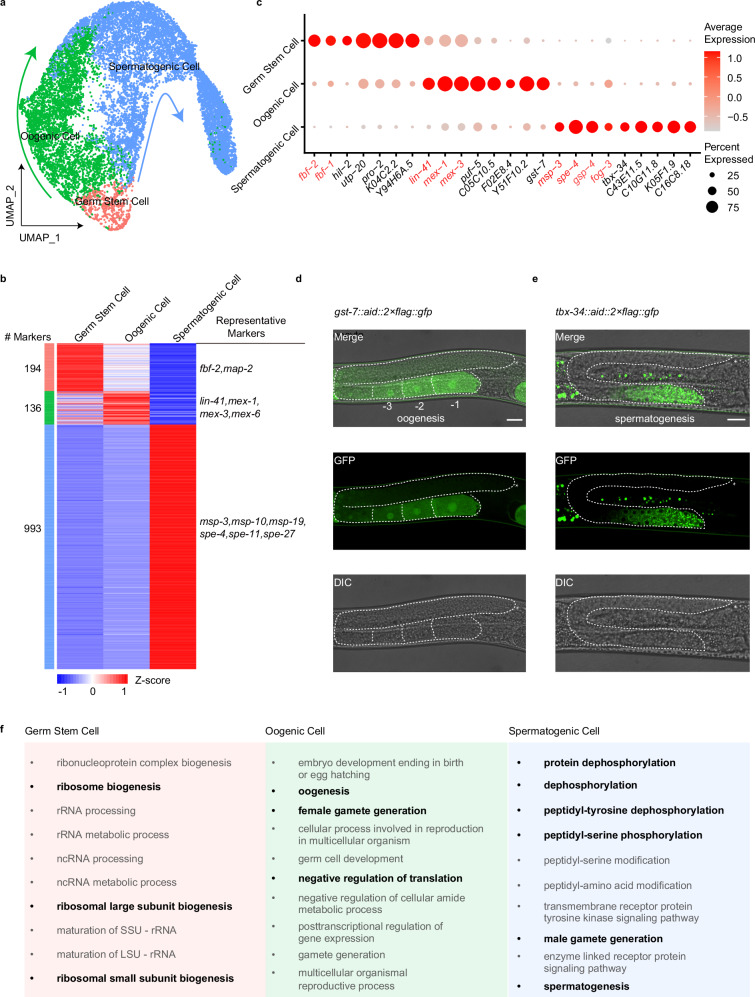
Primary cell types and cellular attributes inferred from scRNA-seq analyses of germ cells in larval 4-stage *C. elegans.* **a** Visualization of major germ cell types from global clustering of all 13,949 cells in UMAP. The fraction of the total cells in each subset is germ stem cell (590), oogenic cell (4474), and spermatogenic cell (8885). **b** Heatmap of marker gene expression in the three cell types. Shown is each gene’s mean expression in each cell type, standardized over the three centroids. Some representative markers are listed. **c** Dot plot showing the relative expression of marker genes in each cell type. The size of each dot refers to the proportion of cells expressing a gene, and the color of each dot represents the calculated scaled expression value; gray is the lowest, and red is the highest. Red marks known makers. Black indicates identified DEGs. **d**, **e** Examples of new marker genes. Visualization of oogenic cell by *gst-7::aid::2×flag::gfp* (adult worm) (**d**). Visualization of the spermatogenic cell by *tbx-34::aid::2×flag::gfp* (L4 worm) (**e**). Scale bars, 20 μm. Stars indicate the distal end of the gonad arm. **f** Enriched GO categories corresponding to DEGs in the cell types in **b**.

Cell clustering analysis, using uniform manifold approximation and projection (UMAP), grouped cells into three distinct populations. We provisionally assigned these clusters to different cell-type identities based on the specific or enriched expression of known cell-type marker genes as follows: germ stem cells (*fbf-1*^*+*^ and *fbf-2*^*+*^), oogenic cells (*lin-41*^*+*^, *mex-1*^*+*^ and *mex-3*^*+*^), and spermatogenic cells (*msp-3*^*+*^*, spe-4*^*+*^*, gsp-4*^*+*^ and *fog-3*^*+*^) (Fig. 1a, c). We observed that there are two differentiation processes of germ cells at the L4 stage, namely oogenesis and spermatogenesis. This observation aligns with our synchronized *C. elegans* L4 stage and with previous studies^35^, which have shown that by the middle-to-late L4 stage, oogenesis begins. Similarly, we observed that additional marker genes are enriched in their respective cell types (Supplementary Fig. S3a). Specifically, *map-2* is enriched in germ stem cells. *Mex-5* and *rme-2* are highly expressed in oogenic cells, and *spe-11*, *spe-27* and *gsp-3* are prominent in spermatogenic cells.

After comparing the transcriptomes within each cluster, we identified 194, 136, and 993 differentially expressed genes (DEGs) among the three cell types and performed Gene Ontology (GO) analysis on these genes (Fig. 1b, f; Supplementary Table S1). Notably, highly DEGs within germ stem cells were associated with ribogenesis, such as ribonucleoprotein complex biogenesis and ribosome biogenesis, as previously reported^36^. However, we observed significant differences between the transcriptional profiles of germ stem cells and those of oogenic and spermatogenic cells. DEGs in oogenic cells were enriched for oogenesis, female gamete generation, and negative regulation of translation. By contrast, DEGs in spermatogenic cells were predominantly associated with male gamete generation and PTMs, such as protein dephosphorylation and phosphorylation. Additionally, we identified potential novel markers from the DEGs and generated GFP transcriptional reporters for genes enriched in specific clusters, enabling direct in vivo examination. GST-7 and C05C10.5 were primarily detected in oocytes (Fig. 1d**;** Supplementary Fig. S3b), while TBX-34 was mainly localized in sperm cells (Fig. 1e), consistent with their mRNA expression.

### Identification of five distinct subpopulations of sperm cells

**Fig. 2:**
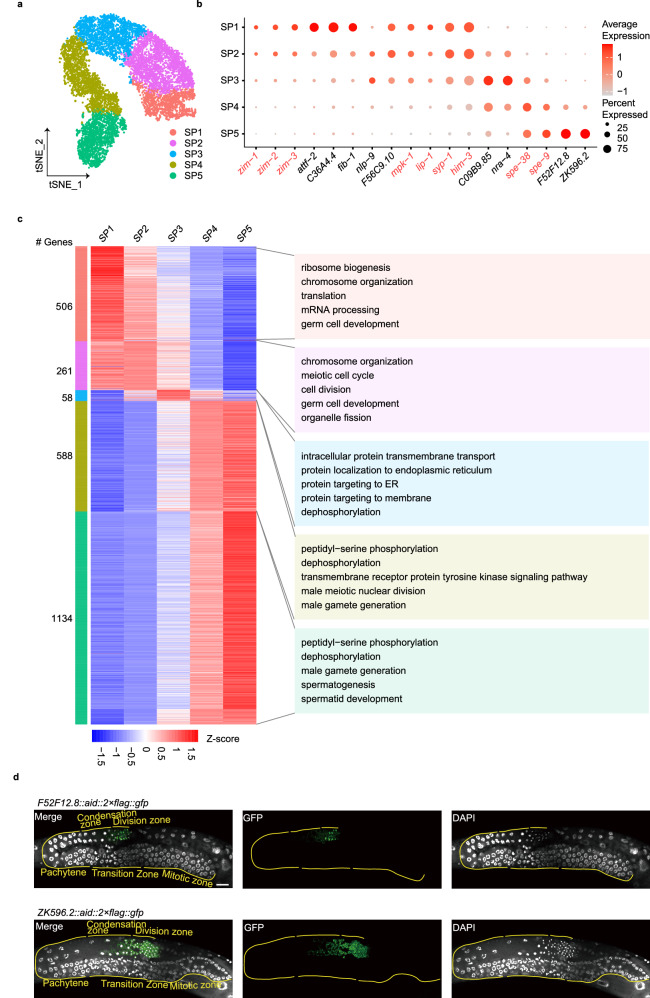
Identification and characterization of spermatogenic cell subpopulations. **a** Re-clustering of the spermatogenic cell, shown in *t*-SNE. The fraction of the total cells in each subset is as follows: SP1 (1210), SP2 (2231), SP3 (1831), SP4 (1992) and SP5 (1621). **b** Dot plot showing the relative expression of marker genes in each inferred cell type. The size of each dot refers to the proportion of cells expressing a gene, and the color of each dot represents the calculated scaled expression value; gray is the lowest, and red is the highest. Red indicates known markers. Black indicates identified DEGs. **c** Heatmap of differential gene expression in the five subsets. Shown is each gene’s mean expression in each cell type, standardized over the five centroids. Shown on the right are representative enrichments of biological processes. **d** Examples of new marker genes. Scale bar, 20 μm.

To investigate the spatiotemporal distribution of gene expression at single-cell resolution during spermatogenesis, we implemented *t*-distributed stochastic neighbor embedding (*t*-SNE) on gene expression data derived from spermatogenic cells and identified five transcriptionally distinct clusters, SP1–5 (Fig. 2a, b; Supplementary Fig. S4). The expression patterns of known marker genes suggested that cluster SP1 (marked by *zim-1*^*+*^, *zim-2*^*+*^, and *zim-3*^*+*^ expression) corresponds to the transition zone preparing for meiotic entry. Clusters SP2–3 (characterized by *mpk-1*^*+*^, *lip-1*^*+*^, *syp-1*^*+*^, and *him-3*^*+*^ expression) represent meiosis I, progressing from prophase I to the pachytene stage. Lastly, clusters SP4–5 (highlighted by *spe-38*^*+*^ and *spe-9*^*+*^ expression) denote the condensation-to-division zone responsible for post-meiotic sperm generation.

Our datasets provided the opportunity to elucidate the exact temporal regulation of signature genes within each cluster during spermatogenesis, detailed as follows. We identified 506, 261, 58, 588, and 1134 DEGs across the five distinct clusters (Fig. 2c; Supplementary Table S2). GO analysis of these DEGs highlighted an enrichment of gene group related to ribosome biogenesis and chromosome organization in the SP1 cluster; chromosome organization and cell division in the SP2 cluster; protein targeting to the endoplasmic reticulum (ER) and dephosphorylation in the SP3 cluster; dephosphorylation/phosphorylation and male meiotic nuclear division in the SP4 cluster; and dephosphorylation/phosphorylation and spermatid development in the SP5 cluster. These results suggest that the early stage of spermatogenesis is controlled by transcription and translation processes, while the later stage is primarily governed by PTMs. Furthermore, our findings reveal a continuous overlap between consecutive clusters, each presenting its unique characteristics during spermatogenesis. Intriguingly, the SP3 cluster, positioned in the midst of these continuously evolving clusters, is distinguished by highly expressed genes that are involved in protein targeting to the ER. For example, the highly expressed genes *sec-61.A* (*Sec61α*), *sec-61.B* (*Sec61ß*), and *sec-61.G* (*Sec61γ*), core components of the translocon, are responsible for translocating newly synthesized proteins into the ER^37^. This translocon is crucial for satisfying the heightened demand for protein synthesis and processing. We hypothesize that the elevated expression of these translocon proteins may set the stage for extensive protein synthesis and prepare for the subsequent cluster cells. In SP5, we also observed 16 DEGs involved in the development of spermatids (Supplementary Fig. S5). Notably, phosphatases GSP-3/4 are reported to regulate the segregation of chromosomes during meiosis, leading to the formation of haploid cells^38^. In summary, our data illuminated distinct cluster-specific molecular events, potentially serving as the driving force behind spermatogenesis.

To further study potential new marker genes, *F52F12.8* and *ZK596.2*, were randomly selected from the identified DEGs, and we employed the CRISPR/Cas9 gene editing technique. We knocked in the AID::2×FLAG::GFP sequence upstream of the stop codon for both *F52F12.8* and *ZK596.2*. Both F52F12.8::AID::2×FLAG::GFP and ZK596.2::AID::2×FLAG::GFP displayed weak expression in the condensation zone, with a significant increase in the division zone (Fig. 2d), which aligns with our scRNA-seq data (Fig. 2b).

### Dynamics of gene expression and regulation along the developmental trajectory of spermatogenesis

**Fig. 3:**
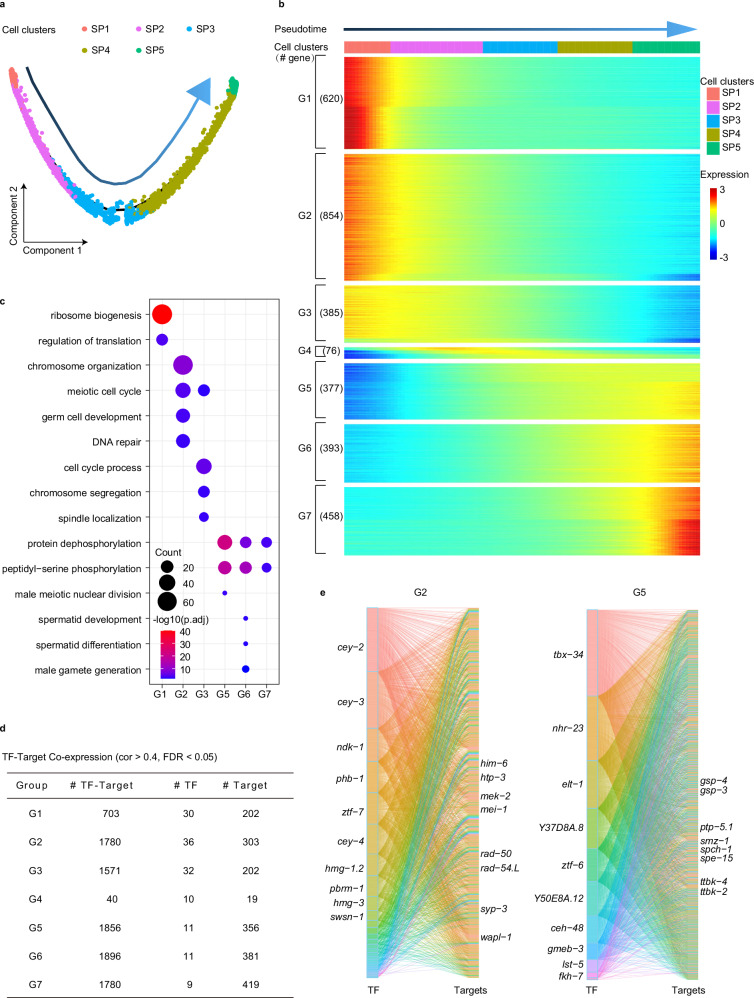
Transcription and regulation throughout spermatogenesis development. **a** Pseudotime trajectory showing the distribution of spermatogenic cells (colored by cell types). The arrow represents pseudotime directions. **b** Heatmap represents gene expression dynamics in sperm cell lineage through the pseudotime. Genes were grouped by similar expression profiles with hierarchical clustering. **c** Results from the GO enrichment test showing the terms associated with the gene categories defined in **b**. **d** The table shows each group’s basic information about the co-expression network. The correlation coefficient between TF and gene greater than 0.4 was considered. **e** Sankey diagram provides insights into the relationships between TFs and genes, where genes are from G2 and G5 (**b**). The left is TFs, and the right is targets. Color represents different TFs. There is a positive relationship between the size of the edges and the correlation coefficient of TFs and targets. TFs are ordered from top to bottom by the number of regulatory targets. Top 10 TFs and some targets are listed.

We performed pseudo-developmental trajectory analysis to further investigate the developmental path of spermatogenic cells in *C. elegans*. The data showed that spermatogenic cells follow a trajectory without branching circuits from the early to late stages of spermatogenesis in pseudotime, revealing that spermatogenesis is a progressive unidirectional differentiation process (Fig. 3a). Additionally, we identified 3163 genes that exhibited dynamic expression over pseudotime (*q* < 0.01) and categorized them into seven groups based on their expression patterns (Fig. 3b; Supplementary Table S3). We estimated the molecular functions of these genes in each group (Fig. 3c). As expected, the functional alterations from group 1 to group 7 (G1–G7) resembled the changes from the SP1 cluster to the SP5 cluster (Fig. 2c), facilitating the exploration of interaction models among TFs and their regulatory genes during spermatogenesis.

TFs play an instrumental role in orchestrating the transcriptional program of spermatogenic cells, which facilitates the production of mature sperm. To investigate TFs and their potential target genes along the developmental trajectory of spermatogenesis, we analyzed the co-expression networks between TFs and different genes from G1 to G7 (Fig. 3d, e; Supplementary Fig. S6 and Table S4). We found that a greater number of TFs may be correlated to gene regulation in the early stages of spermatogenesis compared to the late stages. This suggests that a broader diversity of TFs is required to kick-start the early stages by regulating a variety of cellular processes. By contrast, the later stages of spermatogenic development involve only a handful of TFs. For the target genes in G2, potential regulatory targets such as *him-6*, *htp-3*, *mek-2*, *mei-1*, *rad-50*, *rad-54.L*, *syp-3*, and *wapl-1* have been previously reported to partake in the meiotic cell cycle and chromosome organization^39–46^. In G5, regulatory targets like PP1 phosphatases (*gsp-3/4*) and tau tubulin kinases (*ttbk-2/4*) have been reported as necessary for spermatogenesis^38,47^. These observations suggest that spatiotemporally expressed TFs and their potential regulatory targets participate in specific cellular processes at different stages of spermatogenesis.

### Regulation of kinases and phosphatases by TFs

**Fig. 4:**
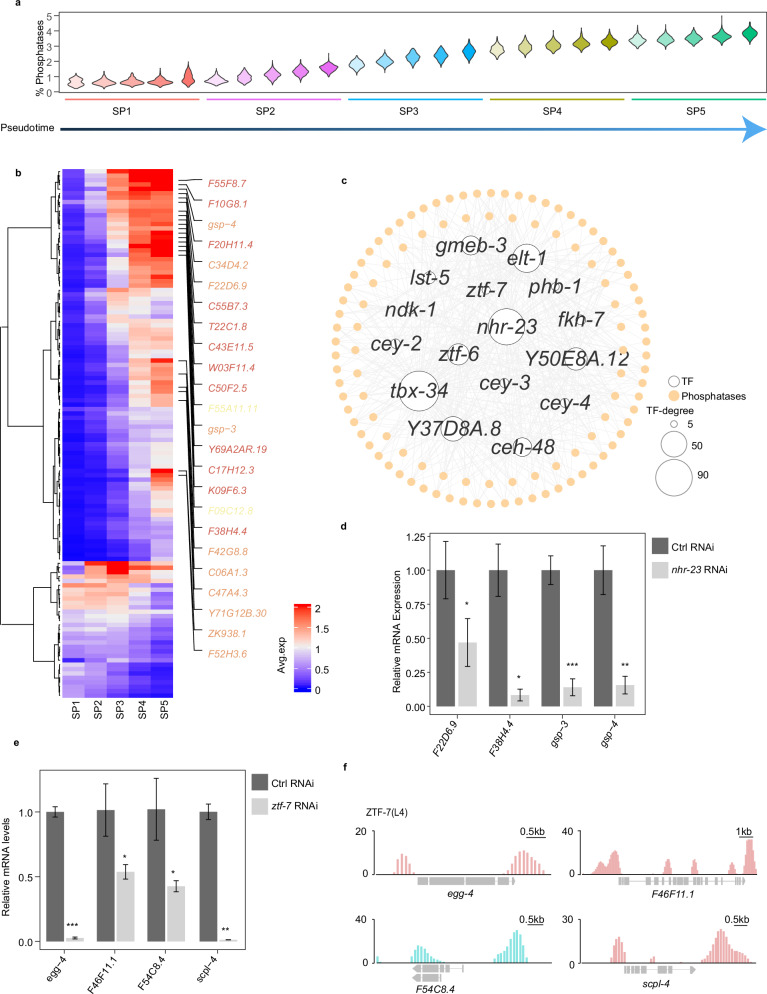
Identification of phosphatase expression patterns and transcriptional regulation. **a** Distribution profile of the percentage of total UMIs from phosphatases per cell across the five subpopulations. Each cell subpopulation is divided into five small subsets along the pseudotime. **b** Heatmap displaying phosphatase expression during spermatogenesis. The mean expression of each gene in each cell type is shown. Only phosphatase genes with average expression levels exceeding 0.5 in at least one of the SP1–5 stages are presented. Some phosphatases highly expressed in the late stage of spermatogenesis are listed. Classification of phosphatases is colored (CC1, Cys-based class I fold (red); PPPL, protein phosphatase-like fold (orange); HP, histidine acid phosphatase fold (yellow)). **c** Transcriptional regulatory network of phosphatases. TFs with larger than 5 edges were considered. The size of the TF nodes is proportional to the number of edges. Hollow circle: TF; solid circle: phosphatases. **d**, **e** qPCR analysis of phosphatases after *nhr-23* RNAi (**d**) and *ztf-7* RNAi (**e**). Graphs represent the relative expression to vector RNAi control. *P < 0.05, **P < 0.01, ***P < 0.001. **f** TF ZTF-7 binds within the promoters of the predictive regulation phosphatases. Data are from the ModERN project.

To further investigate the gene regulatory networks in spermatogenic cells, we concentrated on gene regulation related to phosphorylation signaling, as kinases and phosphatases become increasingly crucial in modulating key events during spermatogenesis^13^. We observed that the proportion of phosphatase and kinase transcripts in the total cellular transcripts progressively increased with the advancement of spermatogenesis, reflecting their escalating importance (Fig. 4a; Supplementary Fig. S7a). Detailed analysis of individual phosphatases and kinases throughout SP1 to SP5 revealed that while a small number of phosphatases and kinases decreased, the majority increased gradually (Fig. 4b; Supplementary Fig. S7b). Our findings bolster the view that phosphatases and kinases serve as pivotal mediators of late spermatogenic cell specification by modulating cellular phosphorylation signaling.

Next, we constructed a co-expression network of phosphatases and kinases to elucidate their transcriptional regulation during spermatogenesis (Fig. 4c; Supplementary Fig. S7c and Table S5). We identified 477 co-expression pairs of TFs and phosphatase genes, involving 16 TFs and 106 phosphatases. Additionally, 568 co-expression pairs were discerned between TFs and kinases, encompassing 15 TFs and 126 kinases. To validate the potential regulatory interactions within the phosphatase regulatory network, TFs are ranked and categorized into three classes based on the number of phosphatases they potentially regulate (Supplementary Table S5). One TF from each class is selected for verification. Specifically, *nhr-23* is predicted to regulate 74 phosphatases, *ztf-6* to regulate 38, and *ztf-7* to regulate 7. Moreover, previous studies have shown that *nhr-23*, *ztf-7*, and *ztf-6*, or their homologs, play roles in cell proliferation^48–50^. We found that inhibition of *nhr-23*, *ztf-7*, and *ztf-6* resulted in a significant decrease in the expression of potentially regulated phosphatases, such as PP1 phosphatases gsp-4 and scpl-4, respectively (Fig. 4d, e; Supplementary Fig. S7d). We also analyzed previously reported ChIP-seq data of ZTF-7^51^ and found that it can bind to the promoter regions of *egg-4*, *F46F11.1*, *F54C8.4*, and *scpl-4* (Fig. 4f). Taken together, these results indicated the spatiotemporal regulation relationships of phosphatases and kinases by TFs in the late stage of spermatogenesis.

### Phosphoproteome identifies the regulatory effect of NHR-23 on phosphorylation

**Fig. 5:**
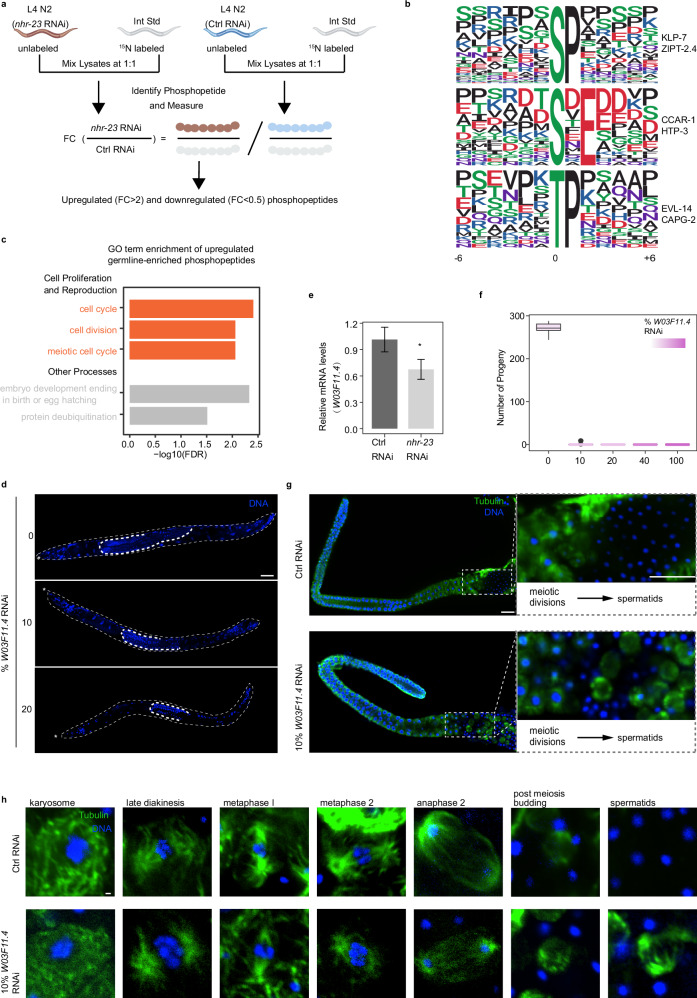
Phosphoproteomic screening identifies the regulatory influence of *nhr-23.* **a** Phosphoproteomics workflow (see Supplementary Fig. S8 for details). **b** The significantly enriched phosphorylation motif from germline-enriched upregulated phosphorylation events. **c** GO term enrichment of germline-enriched upregulated phosphoproteins. Red marks the GO terms related to cell proliferation and reproduction. GO analysis was performed using DAVID. **d** Morphology of *him-8*^*−*^^*/*^^*−*^ adult male under various dosages of *W03F11.4* RNAi. Stars represent the head of the male; scale bar, 30μm. **e** Relative mRNA expression level of *W03F11.4* after *nhr-23* knockdown. *P < 0.05. **f** The number of progeny under different dosages of *W03F11.4* RNAi. **g** Immunostaining of *him-8*^*−*^^*/*^^*−*^ male gonads with vector control RNAi and 10% *W03F11.4* RNAi. Scale bars, 15μm. **h** Immunostaining of *him-8*^*−*^^*/*^^*−*^ male spermatocytes expressing progressing through successive stages of meiotic divisions with vector control RNAi and 10% *W03F11.4* RNAi. Scale bar, 0.5μm.

To determine the regulatory effects of TFs on protein phosphorylation modifications at a proteome-wide scale, we conducted a quantitative phosphoproteomic analysis. *nhr-23* is significantly expressed during the late stages of sperm development, and its knockdown has been shown to result in infertility, suggesting its potentially important role in male reproductive processes^52^(Supplementary Fig. S9a, b). We identified differentially phosphorylated sites in control RNAi- and *nhr-23* RNA interference (RNAi)-treated L4 stage *C. elegans* across three independent biological replicates (Fig. 5a; Supplementary Figs. S8, S9c). Our analysis identified 2709 unique phosphopeptides, corresponding to 866 distinct proteins. Of these phosphopeptides, 2011 were upregulated, and 352 were downregulated (Supplementary Fig. S9d). To explore the phosphorylation changes of proteins in germ cells, we focused on the germline-expressed genes (Supplementary Fig. S8). Our analysis revealed that 253 unique upregulated phosphopeptides, mapping to 115 proteins, and 61 unique downregulated phosphopeptides, mapping to 58 proteins, were identified. In terms of upregulated phosphorylated sites, we found 83.67% were phosphorylated serine (pS), 15.92% were threonine (pT), and 0.41% were tyrosine (pY) (Supplementary Fig. S9e). For the downregulated phosphorylated sites, 63.01% were pS, 35.62% were pT, and 1.37% were pY (Supplementary Fig. S9f).

In order to classify potential substrates, we employed the Motif-X algorithm^53^ to analyze the phosphorylation motifs, using a threshold of *P* < 10^−6^. Predominantly, we identified three phospho-motifs in upregulated phosphopeptides: pSP, pSXE, and pTP (Fig. 5b). The substrate of the pSP motif includes phosphoproteins such as KLP-7 and ZIPT-2.4. KLP-7, a member of the kinesin-13 family, plays essential roles in microtubule regulation during meiotic cell division in *C. elegans*^54^, and the phosphorylation of KLP-7 is necessary for proper microtubule organization^55^. The substrate of the pSXE motif involves phosphoproteins, including CCAR-1 and HTP-3. CCAR-1, a member of the cell division cycle and apoptosis regulator (CCAR) protein family, plays a crucial roles in regulating *C. elegans* germ cell development^56^. The substrate of the pTP motif involves phosphoproteins like EVL-14 and CAPG-2. EVL-14 is crucial for sister chromatid cohesion during meiosis^57^. These results suggest that the TF NHR-23 has a broad role in the regulation of cellular phosphorylation signaling, likely involving the meiotic process.

### Phosphatase regulation by NHR-23 participates in spermatogenic cell division

GO enrichment analysis revealed that germline-enriched phosphoproteins tend to be phosphorylated in the absence of NHR-23, and the most enriched pathways of these upregulated phosphoproteins were related to cell proliferation (Fig. 5c; Supplementary Table S6). Additionally, nearly half of the Protein–Protein Interaction (PPI) components of these phosphoproteins were involved in the cell cycle process, 13 of which were in the meiotic cell cycle (Supplementary Fig. S9g). These findings further suggest that NHR-23 regulates the transcription of phosphatases to participate in the cell cycle process.

To elucidate which phosphatase is regulated by NHR-23 and can affect spermatogenic cell proliferation, we conducted RNAi screening on potential phosphatases within the *nhr-23* gene regulatory network and examined the morphology of *C. elegans* germline (Fig. 5d; Supplementary Fig. S10). Our findings reveal that RNAi of the phosphatase *W03F11.4* led to a shortened germline. Additionally, the expression of phosphatase *W03F11.4* decreased by roughly 30% upon *nhr-23* inhibition (Fig. 5e). *W03F11.4* is specifically expressed in the germline^58^. To assess the influence of a 30% reduction in *W03F11.4* on spermatogenesis, we utilized a gradient RNAi of *W03F11.4* to reduce its expression at different levels. Our results indicate that the repression of *W03F11.4* was RNAi dose-dependent; 10% and 100% RNAi corresponded to ~25% and 85% decreases in *W03F11.4* abundance, respectively (Supplementary Fig. S9h). Notably, even a 10% RNAi can lead to an extension of the L4-to-adult developmental time and infertility (Fig. 5f; Supplementary Fig. S9i). To further investigate this reproductive anomaly, we examined the sperm cell morphology within the germline. We discovered that a 10% RNAi-induced mild reduction in *W03F11.4* can give rise to the retention of substantial quantities of residual bodies in the division zone of spermatogenesis (Fig. 5g). Furthermore, although spermatocytes managed to successfully complete the first meiotic division, they showed a defect in spindle function and post-meiotic budding clearance during the second meiotic process. (Fig. 5h). Thus, phosphatase *W03F11.4* may regulate cell cycle-related protein phosphorylation to facilitate effective spindle progression and clearance of residual bodies. These insights not only establish *W03F11.4* as an example of an *nhr-23*-regulated phosphatase essential for spermatogenesis progression but also propose that protein dephosphorylation and phosphorylation by phosphatases and kinases in spermatogenic cells could represent a crucial post-transcriptional control layer of protein activity during sperm maturation.

### Regulation of small RNA pathways by TFs

**Fig. 6:**
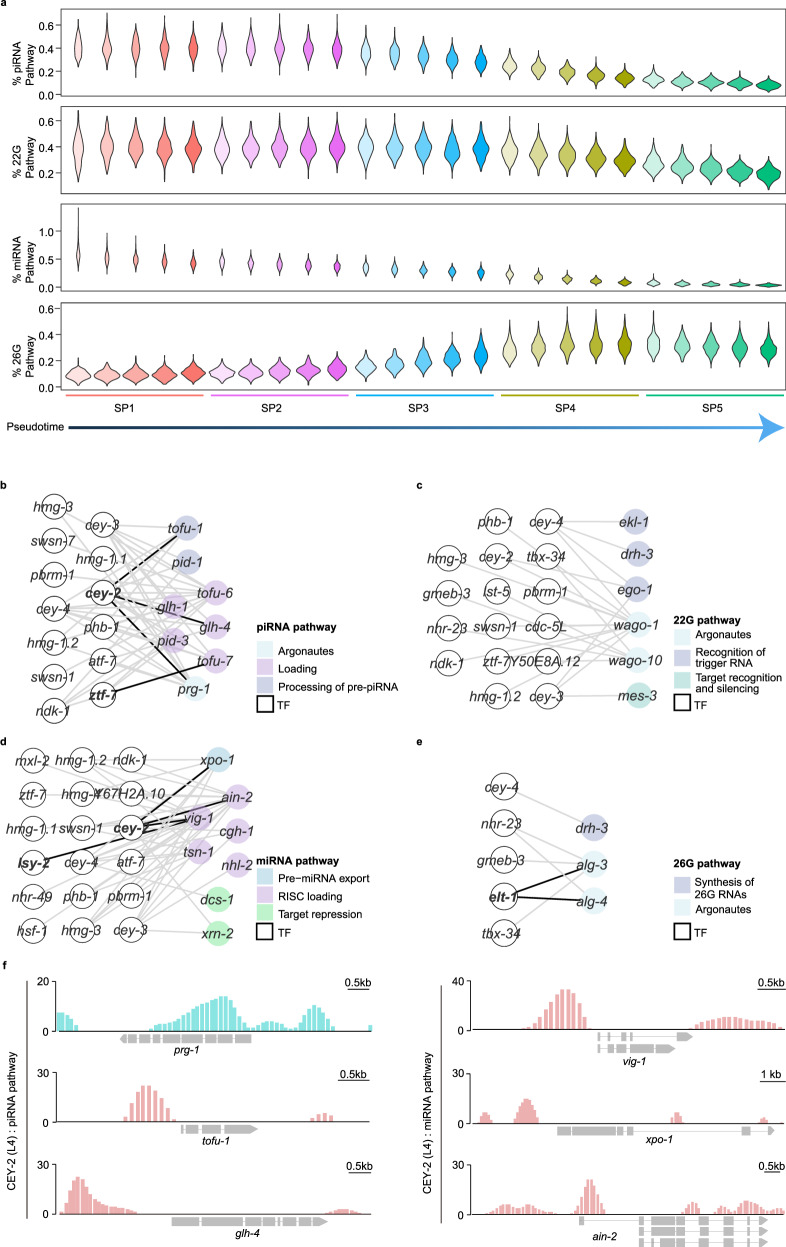
Characterization and regulation of multiple small RNA pathways. **a** Distribution profiles of per-cell attributes compared across the five cell subpopulations. Each cell subpopulation is divided into five small subsets along the pseudotime. From top to bottom: % piRNA Pathway, percent of total UMIs from piRNA pathway components; % 22G Pathway, percent of total UMIs from 22G pathway components; % miRNA Pathway, percent of total UMIs from miRNA pathway components; % 26G Pathway, percent of total UMIs from 26G pathway components. **b**–**e** The small RNA pathways, including the piRNA pathway (**b**), 22G pathway (**c**), miRNA pathway (**d**), and 26G pathway (**e**), are involved in TF regulatory networks. **f** TF CEY-2 binds within the promoters of the components in the piRNA pathway (left) and miRNA pathway (right).

Previous studies underscore the crucial regulatory function of the small RNA pathway during spermatogenesis^59–62^. However, the potential TFs responsible for the production of components in these pathways remain unclear. To address this, we utilized single-cell transcriptome data to analyze dynamic changes in the expression of small RNA pathway components throughout pseudotime as spermatogenic cells developed. Our findings unveiled a progressive decrease in the abundance of transcripts for components in the piRNA pathway, endogenous siRNA 22G pathway (22G production triggered from different resources, including 26G-dependent and piRNA-dependent pathway as well as CSR-1 and RDE-1-associated 22G), and miRNA pathway during spermatogenic cell development (Fig. 6a). piRNA and 22G pathway components were highly expressed in the early stages of spermatogenesis, aligning with previous reports that piRNAs are predominantly present in pachytene spermatocytes^63,64^ (Supplementary Fig. S11a–c). By contrast, the expression of 26G pathway components generally increased as sperm cells matured (Fig. 6a; Supplementary Fig. S11d). This pattern suggests that the activity of the 26G pathway may increase progressively throughout spermatogenesis, becoming increasingly essential for male fertility. Our findings revealed the spatiotemporal expression of different small RNA pathway components at single-cell resolution, offering a comprehensive perspective of small RNA-based regulatory mechanisms during spermatogenesis.

To explore the transcriptional activation of small RNA pathway components, we constructed a co-expression network of these components and their potential TFs during spermatogenesis. We identified 13, 16, 18, and 5 TFs for the piRNA pathway, 22G pathway, miRNA pathway, and 26G pathway, respectively (Fig. 6b–e; Supplementary Table S7). In agreement with previously published TF binding data^51^, we observed an enrichment of the Y-box-binding protein TF, CEY-2, upstream of the transcription start sites of piRNA pathway factors (*prg-1*, *tofu-1*, *glh-4*) and miRNA pathway factors (*vig-1*, *xpo-1*, *ain-2*) (Fig. 6f). Additionally, the zinc finger TFs, LSY-2 and ZTF-7, were found to be enriched upstream of the transcription start site of *vig-1* and *tofu-7* (Supplementary Fig. S11e), respectively. Overall, our data provide an open resource to help decipher the regulation of small RNA pathways and understand their biological functions.

### TF ELT-1 mediates Argonaute proteins ALG-3/4 and their associated siRNA 26G in spermatogenic cells

**Fig. 7:**
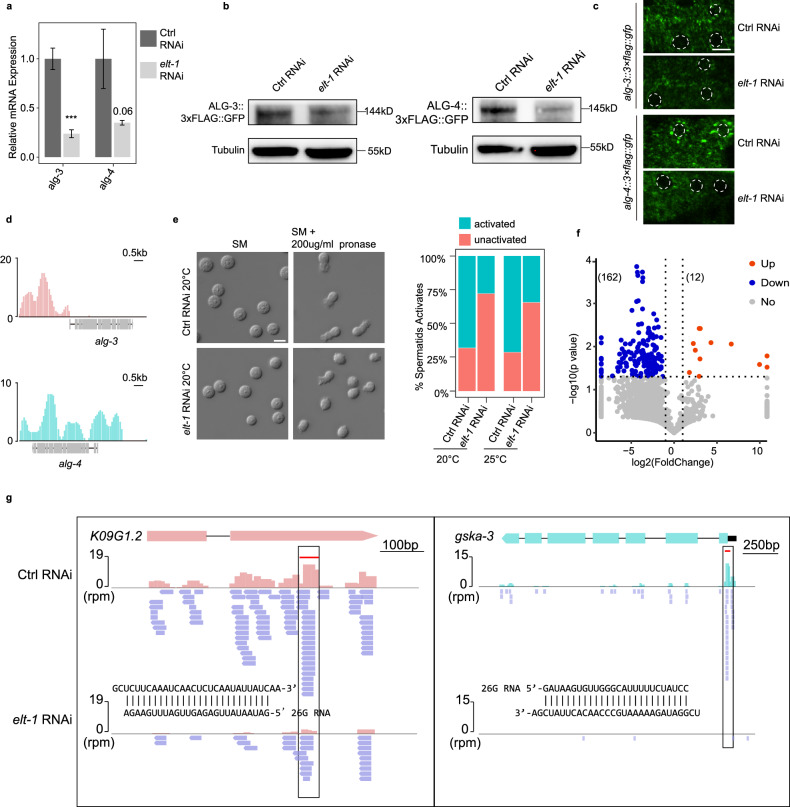
TF ELT-1 regulates the 26G pathway by promoting *alg-3/4* expression. **a** qPCR analysis of *alg-3/4* expression in L4-stage *C. elegans* after knockdown of *elt-1*. Graphs represent the relative expression to vector RNAi control. ***P < 0.001. **b** ALG-3/4 levels were analyzed by western blot assay after *elt-1* RNAi. Antibodies against GFP and tubulin were used. **c** Fluorescent micrographs show GFP-tagged ALG-3/4 expression in the germ cells in RNAi control and *elt-1* RNAi. Scale bar, 5 μm. **d** TF ELT-1 binds within the promoters of *alg-3/4*. **e** Images showing spermatids before and after activation in vector RNAi control and *elt-1* RNAi. The barplot shows statistics of sperm activation. SM: sperm medium. Scale bar, 5 μm. **f** Volcano plot of 26G-RNAs (defined as 25–27nt, with no first nucleotide bias) on *elt-1* RNAi over vector RNAi control. Each dot represents a protein-coding gene. **g** Illustrations for 26G-RNAs distribution along the genes *K09G1.2* and *gska-3*. 26G-RNAs abundance decreased upon *elt-1* RNAi.

To further investigate the regulatory relationship between TFs and components of small RNA pathways, we studied the TF ELT-1 and potential targets ALG-3/4 within the 26G pathway regulatory network (Fig. 6e). ELT-1 is expressed in the germ cells (Supplementary Fig. S12a), and its loss affects sperm functionality^65^, resembling the defective phenotypes observed in the *alg-3*/*4* mutants^62^. These observations led us to ask whether ELT-1 could regulate the expression of *alg-3*/*4*. We used RNAi to suppress *elt-1* expression and found that the mRNA levels of *alg-3*/*4* were downregulated, which was further confirmed by a decrease in the expression of the ALG-3/4 proteins (Fig. 7a–c). Next, we conducted luciferase reporter assays to determine ELT-1’s transcriptional activity on *alg-3*/*4* (Supplementary Fig. S12b). The data revealed that ELT-1 could markedly increase the expression of *alg-3*/*4*. This was further supported by ChIP-seq data^51^ showing that ELT-1 binds to the promoter region upstream of the transcription start site of *alg-3/4* (Fig. 7d). In addition, our result showed that *elt-1* RNAi was indeed phenotypically similar to *alg-3*/4 mutants and resulted in defective sperm activation (Fig. 7e). These findings indicate that ELT-1 activates *alg-3/4* transcription, highlighting the importance of the regulatory network in identifying potential TFs and their targets.

Argonautes ALG-3/4 interact with 26G RNAs to form ribonucleoprotein complexes that protect 26G RNAs from RNase degradation, thereby playing an essential role in the 26G RNA pathway in *C. elegans*^62,66^. We set out to investigate whether inhibition of ELT-1 could also result in the loss of ALG-3/4-associated 26G RNAs. We compared the abundance of 26G RNAs antisense to protein-coding genes before and after *elt-1* RNAi (Supplementary Fig. S12c). We found that there was at least a 2-fold depletion of 26G RNAs among 162 genes, while only 12 genes showed a 2-fold increase of 26G RNAs (Fig. 7f). As illustrated, the antisense 26G RNAs, generated from mRNA template by RNA-dependent RNA polymerase^67^, were significantly diminished along the coding regions of the genes *K09G1.2* and *gska-3* following *elt-1* RNAi (Fig. 7g). These findings suggest that the absence of ELT-1 could lead to the loss of 26G RNAs, possibly due to a lack of protection from Argonautes ALG-3/4. Overall, these results indicate that ELT-1 plays a crucial role in the regulation of the 26G RNA pathway by promoting the expression of *alg-3*/*4* and maintaining 26G RNA species, thus ensuring male fertility.

### A user-friendly web interface designed for navigating the transcriptional atlas of single cells during the spermatogenesis of *C. elegans*

In this study, the single-cell resolution transcriptome data hold significant potential to inform a great deal of additional studies centering on different specific biological questions. To assist researchers in leveraging the single-cell transcriptional atlas of *C. elegans* spermatogenic cells, we have developed an interactive, user-friendly web tool (https://scgerm-atlas.sjtu.edu.cn/website/#/home) (Supplementary Fig. S13). This platform allows users to conveniently access information about their genes of interest. It enables the exploration of gene expression patterns throughout spermatogenesis. Additionally, we provide data on potential relationships between TFs and regulatory genes during spermatogenesis aiding users in understanding the regulatory roles of TFs expressed at different stages of spermatogenic cell development.

## Discussion

The completion of the human genome sequence marked a transition into the post-genomic era for life science research, enabling a comprehensive understanding of gene regulation and expression that are fundamental to biological processes. Previous studies employing single-cell sequencing have predominantly focused on larval development^31^ and aging in adult *C. elegans*^32^, with limited data available on sperm cell development, particularly at the L2 stage when germ cells are just beginning to proliferate. To address this knowledge gap, we obtained cell suspensions from the L4 stage, when most sperm development occurs. Here, we present an RNA map of spermatogenesis using single-cell resolution transcriptome data. The transcriptome data we describe here will facilitate: (1) understanding the spatiotemporal expression of genes and their role in specific biological processes; (2) interpreting the spatial patterns of TFs and potential regulatory genes; (3) a further understanding of how phosphorylation and small RNA signaling pathways create regulatory networks that drive spermatogenesis. We believe that incorporating data from more diverse sources, including tracking the expression of a large number of genes expressed during spermatogenesis, will further contribute to decoding the principles of germ cell differentiation and maturation. Of course, many more diverse exploratory analyses are required, as we have thus far only examined phosphorylation and small RNA signaling pathways. Other aspects, such as the reduction of ribosomal activity for protein translation, regulation of translocon to ER and protein synthesis, DNA damage repair, and more, still necessitate further investigation. However, we hope that the single-cell transcriptome data and transcriptional regulation of gene expression presented in this study can serve as a foundational paradigm to be continually refined and optimized, thereby advancing our understanding of germ-cell differentiation.

The syncytial structure of germ cells is conserved across species^68–70^. In mammals, spermatogonia during mitosis and spermatocytes during meiosis form a syncytial structure connected by stable intercellular bridges, involving hundreds of germ cells^71,72^. Previous studies have mapped the transcriptome of sperm cell development using scRNA-seq, providing a comprehensive analysis of dynamic developmental processes in various species^36,73–76^. Considering the syncytial nature of germlines, which may affect single-cell isolation, we compared our RNA sequencing data from five transcriptionally distinct clusters (SP1–5) with published gene expression data of germline sections (section 1–9 along from distal end to proximal end)^29^ (Supplementary Fig. S14a). We found strong correlations between gene expression profiles at corresponding stages, with coefficients ranging from 0.7 to 0.8 (Supplementary Fig. S14b–f), but not in the non-corresponding stage (Supplementary Fig. S14g). In addition, genes expressed at specific developmental stages in our single-cell data displayed similar functional roles to those identified in the germline sections data (Supplementary Fig. S15). Early spermatogenesis stages are mainly associated with transcription, translation, and meiosis, while later stages involve protein kinases and phosphatases. These findings indicate that our gene expression data are generally consistent with previous studies, likely reflecting physiological conditions. This consistency might imply that the cells obtained in this study were either fully cellularized or remained as nuclei or “cells” with openings connecting to the gonad’s rachis. During germ cell acquisition, we used pronase to digest *C. elegans*. Since pronase can activate spermatids in sperm activation solution^62,77,78^, it may influence gene expression in sperm cells. However, the consistency between our data and previous studies suggests minimal effect, possibly due to using egg buffer instead of sperm activation buffer. Future studies should further optimize germ cell isolation methods.

In the single-cell transcriptome data, a discrepancy exists between the localization of gene transcription and protein expression in the germline. For example, while protein expression of *lst-1* was detected in germline stem cells^79^, its mRNA expression is relatively weak in these cells and higher in other regions (Supplementary Fig. S16a). This transcriptional dynamic pattern is supported by in situ hybridization data from the NEXTDB database^58^ and previously published data on germline gene expression^29^, showing weak *lst-1* expression in germline stem cells but higher expression elsewhere (Supplementary Fig. S16b–d). Future research could further explore the similarities and differences between the transcriptome and proteome in germline cells. Additionally, we compared the number of sperm cells at different developmental stages in single-cell transcriptome data with actual observations. Using chromosome morphology of hermaphrodite L4 larvae^80^, we calculated the number of germ cells at each differentiation stage (Supplementary Fig. S17a) and compared these ratios with sequenced cell proportions (Supplementary Fig. S17b, c). The results showed inconsistencies in stage ratios, with cells in later spermatogenesis stages being relatively easier to obtain, suggesting unequal isolation of germ cells at different stages. Nonetheless, a large number of cells at various stages were obtained, providing a foundation for further analyses.

Single-cell transcriptome profile revealed developmental stage-specific gene expression during spermatogenesis. These specific genes were activated at different stages of sperm development, corresponding to distinct biological events. Moreover, single-cell transcriptome data showed that while each stage of sperm development has unique gene expression characteristics, there is significant overlap in gene expression profiles between successive stages, suggesting that sperm maturation is a sequential, unidirectional, and progressive process, rather than an intermittent and discontinuous cell differentiation process. Simultaneously, we performed a pseudotime analysis of all the germline cells obtained (Supplementary Fig. S18) and discovered that the early-to-middle L4 stage of *C. elegans* is primarily characterized by spermatogenesis. In addition, a subset of oogenic cells was also identified, which might be from the middle-to-late L4 stage of *C. elegans* that have just entered the process of oogenesis. Even though we attempted to synchronize the *C. elegans* to the early-to-middle stage by several rounds of synchronization, a small number of *C. elegans* might still develop to late L4. A more thorough investigation into how oogenesis is initiated and how the dynamic expression of genes drives the whole process of oogenesis needs to be explored in the future.

In the single-cell transcriptome data, we observed an increasing expression of kinases and phosphatases, suggesting their crucial role in the late differentiation stage of sperm cells. During spermatogenesis, chromatin undergoes extensive reorganization, forming a highly compact structure to protect DNA^81,82^. This process renders the DNA inaccessible and may suppress transcriptional regulation in the later stages of spermatogenesis. Posttranslational regulation, including phosphorylation and dephosphorylation, may play a significant role in promoting sperm development during the later stages of spermatogenesis, even when transcription is not occurring. The phosphorylated proteome data showed an involvement of the phosphoproteins in cell division processes and revealed that the TF NHR-23 could regulate the expression levels of kinases and phosphatases, orchestrating the downstream phosphorylation status of numerous proteins. KLP-7 protein, a member of the conserved kinesin family, exhibits kinetochore binding activity and is involved in cellular meiosis^54,55^. EVL-14 protein, a conserved PDS5 cohesion-related factor, is implicated in sister chromatid cohesion during meiosis^57^. ZTF-7, a TF regulates the expression of the conserved protein kinase inhibitor egg-4, which is involved in protein localization in the cell cortex and the organization of the actin cytoskeleton^83^. Much more precise regulatory relationships and molecular functions will require further investigation in the future.

Our work also shows that small RNA signaling pathways undergo dynamic changes during spermatogenesis. The expression of many components in piRNA, endogenous 22G siRNA, and miRNA signaling pathways progressively decreases, whereas the expression in the endogenous 26G siRNA signaling pathway, particularly the key effectors ALG-3/4, gradually increases. We found that the TF ELT-1 can regulate the expression of *alg-3*/*4* in sperm cells. Previous studies have indicated that the 26G siRNA signaling pathway is crucial for sperm maturation, especially under heat stress conditions^62^. Given that this pathway reduces *alg-3/4* expression^66^, which is required for sperm development, we speculate that ELT-1 may control *alg-3/4* expression via regulating the activity of the 26G siRNA pathway. Notably, our study also revealed that the TF NHR-23 can regulate the expression of *gsp-3/4*. Taken together, these results suggest that the expression of *gsp-3/4* may be fine-tuned at multiple layers to ensure its precision. In this study, a minor reduction level of 20% in phosphatase *W03F11.4* was shown to lead to a completely sterile phenotype, underscoring the importance of precise control over phosphatase expression. We speculate that maintaining the appropriate phosphorylation state of downstream targeted proteins by phosphatases or kinases is required to ensure the proper activity of phosphorylated proteins, which is important for proper sperm cell differentiation.

Finally, we developed an interactive data visualization tool to observe the spatiotemporal expression of genes during sperm cell development and identify potential regulatory TFs. To better integrate the original single-cell transcriptome data into the developmental process of sperm cells, we incorporated a 3D display model of the germline, which allows better visualization of the dynamic gene expression data we have obtained.

## Materials and Methods

### Preparation of larval *C. elegans* and enrichment of germ cells

*C. elegans* were cultured on 15-cm plates seeded with the *E. coli* strain OP50. To collect middle L4 stage worms, we used a continuous synchronization method, growing the *C. elegans* on OP50-seeded plates for 48 h at 20 °C. The preparation of single-cell suspensions followed a previously modified method^34^. Synchronized L4 *C. elegans* were collected and washed three times with M9 to remove *E. coli*. The M9 was then removed by washing the pellet three times with sterile ddH_2_O. 160 μL of *C. elegans* pellets was treated with 500 μL of SDS- dithiothreitol (DTT) solution (20 mM HEPES, 0.25% SDS, 200 mM DTT, 3% sucrose, pH 8.0) for 4–5 min. Following SDS-DTT treatment, *C. elegans* were washed five times with 1 mL of egg buffer (118 mM NaCl, 48 mM KCl, 2 mM CaCl_2_, 2 mM MgCl_2_, 25 mM HEPES, pH 7.3, osmolarity adjusted to 340 mOsm with sucrose) and centrifuged at 16,000× *g* for 1 min. *C. elegans* were then incubated in 300 μL of pronase E (15 mg/mL, Sigma-Aldrich P8811, diluted in egg buffer) for 25 min. During the pronase digestion, the centrifuge tubes were placed on a shaker at 1300 rpm. Pronase digestion was halted by adding 700 μL of egg buffer supplemented with 10% FBS, and cells were centrifuged at 9600× *g* for 5 min at 4 °C. The supernatant was removed, and cells were washed twice with 1 mL of egg buffer/FBS per wash. Germ cells were enriched via Percoll density gradient centrifugation. An isodialysis solution was prepared with Percoll and 10× PBS at a volume ratio of 9:1, followed by the preparation of the isodialysis solution with egg buffer at three gradient levels (90%, 70%, and 50%, respectively). We sequentially added 2 mL of the 90% solution, 2 mL of the 70% solution, and 3 mL of the 50% solution into the 15-mL centrifuge tube, then placed the cell suspension on the top layer. After centrifugation at 800× *g* for 20 min, we collected cells from the 90%–70% layer and washed them twice with 5 mL egg buffer/FBS.

### Isolation of germ cells by FACS

We enriched germ cells through density gradient centrifugation and subsequently sorted them using FACS. GFP^+^ germ cells were isolated using a BC MoFlo Astrios with a 70-µm nozzle. Propidium Iodide (PI) was added to the cell sample (final concentration: 4 μg/mL) to label dead and dying cells, aiding in the isolation of viable cells. After sorting, germ cells were examined under a microscope and were observed to be round in shape. Their viability was further assessed using the BIO-RAD TC20 Automated Cell Counter and Trypan Blue staining. When the overall viability of the germ cells exceeded 85%, we proceeded to construct a single-cell sequencing library using cells with a GFP signal and without PI staining.

### scRNA-seq

Germ cells were labeled with sample tags (BD Rhapsody Single-Cell Multiplexing Kit) according to the manufacturer’s guidance. The cell viability was ~89%. Single cells were isolated using Single-Cell Capture and complementary DNA (cDNA) Synthesis with the BD Rhapsody Express Single-Cell Analysis System, and cDNA libraries were prepared using the BD Rhapsody™ Whole Transcriptome Analysis Amplification Kit. Sequencing was conducted on a NovaSeq 6000, and the fastq files were converted using the BD Rhapsody Analysis Pipeline.

### Processing of scRNA-seq data

Reads were mapped to the *C. elegans* reference genome from WormBase WS279. The gene expression matrices were generated using the BD Rhapsody WTA Local bioinformatics pipeline. For quality control, doublets from scRNA-seq data were identified and removed using Scrublet. Cells were retained with 2000–6000 genes, 5000–50,000 unique transcripts, and 0.5%–10% mitochondrial transcripts.

Clustering analyses were adopted using Seurat^84^ (R package V.4.0.3). Highly variable genes were identified, and data were scaled using the SCTransform workflow. Principal component analysis (PCA) was performed using the top 2500 variable genes, and UMAP analysis and plots were generated based on the selected principal components.

For spermatogenic cells, clustering analyses were conducted using Seurat and the LogNormalize workflow to normalize, identify variable genes, and perform scaling of the data. PCA was performed using the top 2500 variable genes. By applying the elbow method, it is determined that the optimal number of clusters for the dataset is five (Supplementary Fig. S4). *t*-SNE analysis and plots were generated based on selected principal components.

### Identification of DEGs

Cell cluster-associated DEGs were identified using the FindAllMarkers function in Seurat. Cell type identities were assigned based on the specific or enriched expression of known cell type marker genes. We required that the expression levels of these marker genes in the target cell type be significantly higher than in other cell types, with an avg_log_2_FC > 0.25.

For the DEGs between germ stem cells, spermatogenic cells, and oogenic cells, the gene expression level was required to be greater than 2, and the *P*_adj_ values were less than 0.05. For the DEGs between each subtype of spermatogenic cells, the expression level was required to be greater than 1.5 and the *P*_adj_ values were less than 0.05. GO analysis of DEGs was performed using the R package clusterProfiler^85^.

### Trajectory analysis

Monocle package^86^ (2.20.0) was used to construct trajectories of spermatogenesis. The expression data were exported from the Seurat object. DEGs were identified by using the differentialGeneTest function in Monocle (*q* < 0.01) and used to order cells in pseudotime along a trajectory. The “DDRTree” algorithm was employed for dimensionality reduction.

### Co-expression networks of TFs

748 TFs of *C. elegans* were obtained from the AnimalTFDB 3.0 database for subsequent analysis^87^. To predict the targets of TF regulation, we calculated the Pearson correlation coefficient between each TF and gene. We only consider relation pairs that exhibited a correlation coefficient greater than 0.4 and *P*_adj_ values less than 0.05. Using these data, we conduct a co-expression regulatory network analysis of TFs using Cytoscape 3.9.1^88^.

### Lifespan analysis

Lifespan assay was performed using synchronized worms maintained at 20 °C on nematode growth medium (NGM) plates seeded with *E. coli* OP50. 20 adult worms were used to evaluate lifespan. The Kaplan–Meier method^89^ was used to estimate the survival of worms, and differences in survival were evaluated by a log-rank test.

### Brood size analysis

All brood size assays were performed at 20 °C using synchronized worms, with 20 worms counted. The number of offspring during the reproductive period was measured. Adult worms were transferred to a new plate every 24 h, and the hatched eggs on the previous plate were counted. This process was repeated until the number of offspring hatched within 5 days was determined. Statistical analysis was performed using a Wilcoxon rank sum test.

### Phosphoproteomics and data analysis

For phosphoproteomics, total protein was extracted from L4 stage worms under each test condition using RIPA lysis buffer supplemented with protease and phosphatase inhibitors. Subsequently, samples were mixed at a 1:1 ratio with ^15^N labeling proteins as a reference^90^. Following DTT reduction and iodoacetamide alkylation, the combined samples were digested with trypsin. Phosphopeptides were enriched from total peptides using the Fe-NTA Phosphopeptide Enrichment Kit (ThermoFisher Scientific, A32992). The enriched phosphopeptides were separated using the Thermo Vanquish Neo integrated nano-HPLC system, which was directly interfaced with a Thermo Exploris 480 mass spectrometer equipped with FAIMS Pro for final protein identification. The resulting data were analyzed using pFind by comparing MS/MS spectra with the Uniprot *C. elegans* protein database. For the phosphoproteome, default parameters were set for protein identification. Peptides with distinct amino acid sequences and modifications were considered unique. For quantification, only unique peptides were accounted for. The assay was performed on three biological repeats, and at least two identified phosphopeptides were required. The relative abundance of individual unlabeled phosphopeptides was determined using a ^15^N-labeled sample as an internal standard. Phosphopeptides with a fold-change ratio > 2 or < 0.5 were classified as differentially expressed phosphopeptides. The Momo and motif-x software algorithms were used to analyze the motif characteristics of modification sites^53,91^. The analysis focused on the peptide fragment sequence consisting of 6 amino acids upstream and downstream of each identified modification site. The protein sequences in the database were used as background parameters, with other parameters set to default. A sequence form was considered a motif of modified peptide segments when the number of peptide segments in that form exceeded 20, and the statistical test *P* value was less than 0.000001. GO analysis of differentially expressed phosphoproteins was performed using DAVID (FDR < 0.05).

### Gene sets

The gene set for kinases was obtained from Wormbook^92^, while the gene set for phosphatases was derived from Hema Bye-A-Jee’s studies^93^. The gene sets related to endogenous small RNA pathways, such as the 22G pathway and 26G pathway, were sourced from Wormbook^94^. The information regarding miRNA-centered complexes was obtained from the work of Victor Ambros’ lab^95^ and Wormbook^94^. Additionally, the components involved in the piRNA pathway were identified from these studies^94,96,97^.

### Small RNA library preparation and analysis

Small RNAs were isolated from total RNAs extracted from prepared worm samples using the mir-Vana miRNA isolation kit (ThermoFisher Scientific, AM1560). The small RNA cloning procedure was performed as described previously^98^. Briefly, RNA 5′ Pyrophosphohydrolase (RppH) (NEB, M0356S) was used to trim the 5′ triphosphate of endogenous 22G-RNAs to monophosphate. The 3′ and 5′ adapters were sequentially ligated using truncated ligase 2 (NEB, M0373S) and ligase 1 (NEB, M0437M). cDNA was synthesized using Superscript III reverse transcriptase (Invitrogen, 18064022) and amplified by Q5 polymerase (NEB, M0491S), with adapter sequences added during this step. Amplified PCR products were subjected to 12.5% native PAGE for size selection. High-throughput sequencing was then performed using multiple Illumina platforms.

Adapter sequences in the raw reads were trimmed using Cutadapt (version 2.5), with the adapter sequence “AGATCGGAAGAGCACACGTCTGAACTCCAGTCAC” as a reference. Reads were then mapped to the *C. elegans* genome (WormBase release WS279) using the STAR-aligner (version 2.5.2b). Read counts were obtained using a custom R script and normalized to the library size (reads per million, RPM). To delineate a set of 26G-targeted genes, which consist of 25–27 nt RNAs (26G RNAs) that are antisense to protein-coding genes, we established a cutoff that included genes with either a 2-fold enrichment or depletion, and a *P* value of less than 0.05, as determined by the Student’s *t*-test.

### *C. elegans* maintenance and CRISPR/Cas9-mediated genome editing

Following established standard protocols^99^, all *C. elegans* strains were maintained at 20 °C on NGM plates seeded with *E.coli* OP50. We used the WT N2 strain. When constructing strains with endogenous fluorescent tags, we utilized the CRISPR/Cas9 editing strategy^100^. To improve the efficiency of editing, we selected two sgRNAs for each gene. The donor plasmid was generated through Gibson assembly, comprising the up arm, tag sequence, and down arm in sequence. We mixed ordered Cas9 protein from IDT with sgRNA, the donor, and the *rol-6* marker plasmid, and injected this mixture into the germline of young adult *C. elegans*. Transgenic strains were selected based on the *rol-6*-related roller phenotype^101^ and then genotyped. The details of the genes are presented in Supplementary Tables S8, S9.

### Western blot analysis

Synchronized L4 *C. elegans*, treated with RNAi, were homogenized in SDS lysis buffer and heated at 95 °C for 10 min. The lysates were centrifuged at 12,000 rpm for 10 min to collect the protein supernatant. Proteins (100 μg) were separated by SDS-PAGE and then transferred to PVDF membranes (BioRad). For blot probing, a mouse anti-GFP antibody (Sigma, 1:5000) and an HRP-conjugated goat anti-mouse IgG (Immunology, AE012, 1:10,000) were used.

### RT-qPCR

400 ng of total RNA was used as the template for the first-strand cDNA synthesis, employing the 1st Strand cDNA Synthesis SuperMix (Novoprotein, E047). Real-time PCR was performed using the SYBR qPCR Master Mix (Vayzma, Q711) on the Roche LightCycler® 480 Real-Time PCR Detection System. The primer sequences of the target genes are provided in Supplementary Table S10.

### Additional resources

Our online tool CelegansGermAtlas can be accessed via the following URL: https://scgerm-atlas.sjtu.edu.cn/website/#/home. The source code for the website is available on GitHub: https://github.com/CMACH508/scgerm-atlas.

## Supplementary information

Supplementary Information

Table 1, Table 2, Table 3

Table 4

Table 5

Table 6

Table 7

## Data Availability

scRNA-seq data and small RNA sequencing data are available in the NCBI GEO database with accession numbers GSE274290 and GSE274291, respectively.
