# Supplementary material for: Spatiotemporal single-cell architecture of gene expression in the Caenorhabditis elegans germ cells
Source: Cell Discov. 2025 Mar 18;11:26. doi: 10.1038/s41421-025-00790-4 (PMC11914268; doi:10.1038/s41421-025-00790-4)

Supplementary Fig. S1 Progeny and lifespan analysis of strains with labeled germ cells and developmental synchronization.

a The progeny number for different strains. PRG-1, GLH-1, and PGL-1 were well-known protein markers, only expressed in the germ cells. P value: ***p<0.001, ns p>0.05.

b Lifespan analysis for each strain (log-rank test, n = 20 animals per strain).

c The ratio of different larval stages for *gfp::tev::flag::prg-1* strain from 40.5 hours to 48 hours following multiple life-cycle synchronizations. L4 indicates the fourth larval stage.

Supplementary Fig. S2 Isolation of germ cells from L4 *C. elegans* for scRNA-seq analysis.

a Schematic of the experimental workflow for isolating germ cells.

b Germ cells were enriched by density gradient centrifugation. Scale bar, 50μm.

c Sorting of PI-negative and GFP-positive germ cells by FACS using the enriched germ cells (**b**).

d Image of the sorted germ cells as indicated in (**c**). Scale bar, 50μm.

Supplementary Fig. S3 a Dot plot showing the relative expression of genes. **b** Expression pattern of new marker gene.

Supplementary Fig. S4 The elbow method is used to determine the optimal number of clusters.

Supplementary Fig. S5 Cluster SP5-enriched genes related to spermatid development.

a Heatmap of spermatid development-related genes that are enriched in cluster SP5.

b Diagram of spermatogenesis. As illustrated, the potential regulated genes are indicated in red letters.

Supplementary Fig. S6 Regulatory network along the developmental trajectory of sperm cells. From left to right, Sankey diagram provides insights into the relationships between TFs and genes, where genes are from G1, G3, G4, G6, and G7.

Supplementary Fig. S7 Expression patterns of kinases and transcriptional regulation.

a Distribution profile of per-cell percentages of total UMIs from kinases across the five cell subpopulations. Each cell subpopulation can be further divided into five small subsets along the pseudotime.

b Heatmap illustrating the expression levels of kinases during spermatogenesis. The mean expression of each gene in each cell type is shown. Average kinase expression levels above 0.5 in at least one of SP1-5 stages are presented. Some kinases highly expressed in the late stage of spermatogenesis are listed. Classification of kinases is colored (TK, tyrosine kinase (red); CK1, casein kinase 1 (orange); CAMK, Ca^{2+}/calmodulin-dependent kinase (purple); others (yellow)).

c Transcriptional regulatory network of kinases. TFs with larger than 5 edges were

considered. The size of the TF nodes is proportional to the number of edges. Hollow circle: TF; solid circle: kinases.

d qPCR analysis of phosphatases after *ztf-6* RNAi. Graphs represent the mRNA expression, relative to vector RNAi control. P value: *p<0.05, **p<0.01, ***p<0.001.

Supplementary Fig. S8 Phosphoproteomic detailed workflow.

Supplementary Fig. S9 *nhr-23* regulates the phosphorylation of proteins.

a Expression patterns of TF *nhr-23* during spermatogenesis.

b The progeny number of worms with control and *nhr-23* RNAi treatment.

c The PCA is performed using Phosphoproteomic data.

d Dot plots of phosphopeptides levels. Dashed lines denote regions where loci exhibit a 2-fold change in phosphopeptides levels compared to the vector RNAi control.

e The distribution of the phosphorylated serine (pS), threonine (pT), and tyrosine (pY) among the identified germline-enriched upregulated phosphorylation events.

f The distribution of the phosphorylated serine (pS), threonine (pT), and tyrosine (pY) among the identified germline-enriched downregulated phosphorylation events.

g Interaction network of proteins harboring phosphorylation sites that are germline-enriched upregulated when compared to the vector RNAi control. Interactions were established by using the STRING database and visualized by Cytoscape. The orange color indicates that the proteins are involved in cell cycle. The red circle indicates that the proteins are also involved in meiotic cell cycle. The gray shows other proteins.

h *W03F11.4* relative mRNA expression level after different gradient concentrations of *W03F11.4* RNAi. P value: **p<0.01.

i The duration required for *C. elegans* to reach each developmental stage under various dosages of *W03F11.4* RNAi.

Supplementary Fig. S10 Male germline morphology screen following phosphatase genes RNAi.

Shown are the *nhr-23-mediated* phosphatases with the top 20 correlation coefficients. Scale bar, 10μm.

Supplementary Fig. S11 Expression pattern of components in the piRNA pathway, 22G pathway, miRNA pathway, and 26G pathway.

a The left panel presents a heatmap of piRNA pathway components expressed during spermatogenesis, while the right panel illustrates the piRNA pathway.

b The left panel shows a heatmap of 22G pathway components' expression during spermatogenesis, and the right panel depicts the 22G pathway.

c On the left is the heatmap of miRNA pathway components that are expressed in spermatogenesis. The right is the miRNA pathway.

d The left is the heatmap of 26G pathway components' expression in spermatogenesis. The right is the 26G pathway.

e TFs bind within the promoters of the components *vig-1* and *tofu-7* in miRNA and piRNA pathways.

Supplementary Fig. S12 TF *elt-1* regulates Argonautes *alg-3/4* expression and 26G small RNAs.

a Expression patterns of TF *elt-1* during spermatogenesis.

b Assay for transcriptional activity of *elt-1* through luciferase activity. P value: $^{**}p<0.01$, $^{***}p<0.001$.

c Heatmap shows repeatability between small RNA-seq samples.

Supplementary Fig. S13 A user-friendly interface for investigating spatial expression during the spermatogenesis of *C. elegans*

a The transcriptional profiles of genes in the germline of the L4 stage are displayed, with *zyg-11* serving as an exemplar.

b A three-dimensional transcriptional atlas of the gene is provided, using *zyg-11* as an illustrative example.

c, d The expression pattern of genes along sperm cells (**c**) and pseudotime trajectories (**d**) are presented, exemplified by the gene *zyg-11*.

e Information on potential interactions between transcription factors and genes during spermatogenesis is provided. Relevant results can be identified by searching with the specific transcription factor and target gene of interest.

Supplementary Fig. S14 Single-cell gene expression data were compared with existing germline gene expression data.

a Illustration of the *C. elegans* male gonad.

b-f Comparison of single-cell gene expression data with gene expression data from male gonad sections at the same developmental stage.

g Comparison of single-cell gene expression data with gene expression data from male gonad sections at the different developmental stages. Single-cell gene expression data are shown in blue and the male section data is shown in red.

Supplementary Fig. S15 Genes expressed at specific developmental stages in single-cell data show similar functional roles to those identified in the germline sections. The top panel depicts the male germline sections, while the bottom panel shows the single-cell data. Cartoons of gonads with red indicate regions of high gene expression, and the corresponding GO terms are displayed on the right.

Supplementary Fig. S16 The expression of *lst-1* in different data.

a The expression of *lst-1* in single-cell transcriptome data.

b In situ hybridization of endogenous transcripts of *lst-1* at the L4 stage, and are taken from the Nematode Expression Data Base (https://nematode.nig.ac.jp/). Red stars indicate the distal end of the gonad arm.

c Illustration of the *C. elegans* male gonad.

d Dynamic expression of *lst-1* in male gonad.

Supplementary Fig. S17 Comparison of the number of spermatogenic cells at

different stages of differentiation.

a The number of germ cells at each stage of differentiation in hermaphrodite L4.

b The percentage of germ cells at various stages of sperm development during the L4 stage.

c The data predicted the proportion of cells at each stage of spermatogenesis.

Supplementary Fig. S18 Pseudotime trajectory showing the distribution of germ cells.

Supplementary Table S1. Marker genes for each cell cluster.

Supplementary Table S2. Marker genes for each cell cluster during spermatogenesis.

Supplementary Table S3. A list of genes clustered based on similar expression profiles across pseudotime.

Supplementary Table S4. The regulatory interactions between TFs and genes in G1-7.

Supplementary Table S5. Transcriptional regulation of phosphatases and kinases.

Supplementary Table S6. Phosphoproteomic data following *nhr-23* RNAi.

Supplementary Table S7. Transcriptional regulation of small RNA pathways.

Supplementary Table S8. *C. elegans*' strains, Related to methods

Genotype Methods	Subject to	Methods/reference
N2		CGC
gfp::tev::flag::prg-1		Shen et al., 2018
gfp::glh-1		Dai et al., 2022
pgl-1::mrfp		Shen et al., 2018
gst-7::aid::2×flag::gfp	N2	CRISPR
tbx-34::aid::2×flag::gfp	N2	CRISPR
C05C10.5::aid::2×flag::gfp	N2	CRISPR
F52F12.8::aid::2×flag::gfp	N2	CRISPR
ZK596.2::aid::2×flag::gfp	N2	CRISPR
alg-3::3×flag::gfp		Lei Liu et al., 2023
alg-4::3×flag::gfp		Lei Liu et al., 2023
him-8-/-	N2	CRISPR

Supplementary Table S9. CRISPR Oligos, Related to methods

Name	Sequence	Gene
gst-7_sgRNA1	*atttggaaatcatTTAGAA*	*gst-7*
gst-7_sgRNA2	*tcatTTAGAATGGAGTAAC*	*gst-7*

tbx-34_sgRNA1	*tgtccggtcccatCTATTG*	*tbx-34*
tbx-34_sgRNA2	*ACGCCACAATAGatgggac*	*tbx-34*
C05C10.5_sgRNA1	*atctgtctacatgattccc*	*C05C10.5*
C05C10.5_sgRNA2	*aatcaatcttcagacaggt*	*C05C10.5*
F52F12.8_sgRNA1	*CATAAGCTTCGAGTTGTCT*	*F52F12.8*
F52F12.8_sgRNA2	*GCATAAGCTTCGAGTTGTC*	*F52F12.8*
ZK596.2_sgRNA1	*AACGAGTTTCCTTATGATT*	*ZK596.2*
ZK596.2_sgRNA2	*GAACGAGTTTCCTTATGAT*	*ZK596.2*
him-8_mutant_sgRNA	*aattgctggaagaggaaata*	*him-8*

Full-length sequences of *gst-7_aid-2×flag-gfp*_donor   Red: insertions

actctaggcagtttgacacctagagatgtaaacaaagtgcttggtatgactgagagatagaagtttggagtcaatgattgttta ctcaaagaaaactttgaactttaggttttaattatattatttttcagGCACTCCATTCGGACAGCTTCCACTCCT TGAAGTTGACGGAAAGGTTCTTGCCCAATCTCATGCTATCGCCCGTTACTTGGCTCG TCAGTTCGGAATCAATGGAAAGTGTGCATGGGAGGAGGCTCAAGTCAACTCGGTTG CTGATCAATTCAAGGATTACCTCAACGAAGTTCGTCCATACTTCATGGTGAAGATGGG ATTTGCTGAAGGAGATCTCGATGCTCTTGCCAAGGACGTCTTCCTTCCAGGATTCAA GAAGCACTATGGATTCTTTGCTAACTTCCTCAAGTCGGCTGGATCCGGATACTTGGTT GGAGACTCTTTGACCTTTGTCGACTTGCTCGTCGCTCAGCACACTGCTGATCTTCTG GCTGCCAACGCAGCTCTTCTCGATGAATTCCCACAATTCAAGGCTCATCAGGAAAAG GTTCACTCGAATGCCAACATCAAGAAGTGGTTGGAGACTCGTCCAGTTACTCCATTCc ctaaagatccagccaaacctccggccaaggcacaagttgtgggatggccaccggtgagatcataccggaagaacgtgat ggtttcctgccaaaaatcaagcggtggcccggaggcggcggcgttcgtgaagGATTACAAGGATGACGACGA TAAGGATTACAAGGATGACGACGATAAGGAGAACCTCTACTTCCAATCGATGAGTAAA GGAGAAGAACTTTTCACTGGAGTTGTCCCAATTCTTGTTGAATTAGATGGTGATGTTA ATGGGCACAAATTTTCTGTCAGTGGAGAGGGTGAAGGTGATGCAACATACGGAAAAC TTACCCTTAAATTTATTTGCACTACTGGAAAACTACCTGTTCCATGGgtaagtttaaacatatat atactaactaaccctgattatttaaattttcagCCAACACTTGTCACTACTTTCTGTTATGGTGTTCAAT GCTTCTCGAGATACCCAGATCATATGAAACGGCATGACTTTTTCAAGAGTGCCATGCC CGAAGGTTATGTACAGGAAAGAACTATATTTTTCAAAGATGACGGGAACTACAAGACA CgtaagtttaaacagttcggtactaactaaccatacatatttaaattttcagGTGCTGAAGTCAAGTTTGAAGG TGATACCCTTGTTAATAGAATCGAGTTAAAAGGTATTGATTTTAAAGAAGATGGAAACA TTCTTGGACACAAATTGGAATACAACTTCAACTCACACAATGTATACATCATGGCAGAC AAACAAAAGAATGGAATCAAAGCTgtaagtttaaacatgattttactaactaactaatctgatttaaattttcagA ACTTCAAAATTAGACACAACATTGAAGATGGAAGCGTTCAACTAGCAGACCATTATCA ACAAAATACTCCAATTGGCGATGGCCCTGTCCTTTTACCAGACAACCATTACCTGACC ACACAATCTGCCCTTTCGAAAGATCCCAACGAAAAGAGAGACCACATGGTCCTTGTT GAGTTTGTAACAGCTGCTGGGATTACACATGGCATGGATGAACTATACAAATAAatgattt ccaaattcacatggtctctttatgtatttttgtattgatatgggataaatgattgcattgaaatctatttattctgaaaagttctctaaaa aataagatagcagaagcaaccgatatatttgttcagacatccgaatcatcatgaaaaggcactttttgaactgagttgtcagta acatctgcgcagtgggaagagtaaatgatgacaaactttaaagtttccaatatactctttattttaatgtacatttatctaattcaac cagttcattgaaacagacataacgccaaactatatttgtggtttgttgtcagcagagtgcgatcaaagcagtgatgacttaaca gtacacacacacttagagaatatgagtataaatagaaacttcttggtttttatttcaaactgtatcacagacttgtactccccaatt acttgaaaagtggtagtaataatggtccactacaagctatcatatttccccatccgtggagctggagaggttattcgacagata

tttgtatatgctggacaaagc

Full-length sequences of *tbx-34_aid-2×flag-gfp*_donor **Red: insertions**

ACGTCACCACAGGAAAATCGACGGAATTCCAGTTCCAACAAATGGAATTCATCGCCG
TCAAGTCCTACCAATCGGCGCGAATTCGCCACACAAAACGTGCACCGCGAAAGATG
AATCTTGCTCCGGGTAGTTCGCAGAAGCCACAACTCATTGTACCGGACATTCTCCAC
TCGCCCACCTATGGCTTCACCGCGGCGCCACCACCGTTCCCGTTCGAGTACTGGCT
TTTGTACCCACAAATTCAATATCAGATTCAGCAGCTCGCGTACTCACTTCCAATGGGA
CCACCGATGGTTCCAATTCACCATATTCAATGCACTGAGGCTTCTCAGCGTGTCTATG
CTCCAGAGTACGGATGGAATTCGATTCTTATGCCGGGAGCTCACAAAGAGCAAGATG
ATCATTACATGTTTCACCATGAAATTTGGGCACCGCAGGACCACGAACTCGAGAAGC
TGCATGTGCTCACTGAACAGgtttttaccgaactctttacctgaaatttatttgaacgttccagAAAAATAAGC
CCTCGGAGACGCCACAAcctaaagatccagccaaacctccggccaaggcacaagttgtgggatggccaccg
gtgagatcataccggaagaacgtgatggtttcctgccaaaaatcaagcggtggcccggaggcggcggcgttcgtgaagG
ATTACAAGGATGACGACGATAAGGATTACAAGGATGACGACGATAAGGAGAACCTCTA
CTTCCAATCGATGAGTAAAGGAGAAGAACTTTTCACTGGAGTTGTCCCAATTCTTGTT
GAATTAGATGGTGATGTTAATGGGCACAAATTTTCTGTCAGTGGAGAGGGTGAAGGT
GATGCAACATACGGAAAACTTACCCTTAAATTTATTTGCACTACTGGAAAACTACCTGT
TCCATGGgtaagtttaaacatatatatactaactaaccctgattatttaaattttcagCCAACACTTGTCACTACT
TTCTGTTATGGTGTTCAATGCTTCTCGAGATACCCAGATCATATGAAACGGCATGACTT
TTTCAAGAGTGCCATGCCCGAAGGTTATGTACAGGAAAGAACTATATTTTTCAAAGAT
GACGGGAACTACAAGACACgtaagtttaaacagttcggtactaactaaccatacatatttaaattttcagGTGC
TGAAGTCAAGTTTGAAGGTGATACCCTTGTTAATAGAATCGAGTTAAAAGGTATTGATT
TTAAAGAAGATGGAAACATTCTTGGACACAAATTGGAATACAACTTCAACTCACACAAT
GTATACATCATGGCAGACAAACAAAAGAATGGAATCAAAGCTgtaagtttaaacatgattttactaa
ctaactaatctgatttaaattttcagAACTTCAAAATTAGACACAACATTGAAGATGGAAGCGTTCAA
CTAGCAGACCATTATCAACAAAATACTCCAATTGGCGATGGCCCTGTCCTTTTACCAG
ACAACCATTACCTGACCACACAATCTGCCCTTTCGAAAGATCCCAACGAAAAGAGAG
ACCACATGGTCCTTGTTGAGTTTGTAACAGCTGCTGGGATTACACATGGCATGGATG
AACTATACAAATAGatgggaccggacaattttactcataaaatattcgagataaatgtttttgaaaaggatttttttattgtg
ctgacaatgataaacatatttgcaggacactggcgaaaatcgcaaataaagtgccgaaagtcgccggcaattgacagaa
aattgctgatttttgtttttaagattttagctaaaattatcgtaatttgttcgaaattttaaaccccccataaaaatttcgatcatttgag
cacatttgttttaattctacagtttgctcattcattttagaattttcaaactttacttgtagaagataccgttcttataattaattttttaaatt
ttatttttaatgcgaaaattattgaactgtccagaacttttcagaaatttttagcattttctagaacattcaaatgtatccaaaattttcc
aatactttgcagaactttctcgaaagttttcaaaattccttgataccatcacaaataattttttttttcttcaaactacagtaatattata
gtaatataagtaatataagatccgacaaagttaaacttttcaatgtagtatgttaacttttatagaagagtatgaaattatagaaa
aatttaaaatccctagttattgagttcttgccac

Full-length sequences of *C05C10.5_aid-2×flag-gfp*_donor **Red: insertions**

accagacgtcagaagagcgatcagacggacaccgccgccacgtgtcaaccgtgtggctccgattcgacttccagagcgg
cgtccattgcggcaattgaacgccaatgacgccagaagaccagcacaagacgctgcaaatcgcataaatcaagagaat
atgcgtgcaggaaatatcgacaatgggcagccacgtcgtcgagcactctccccgacacaattgccaggagttcgtgcgatt
ccaaaggacttcaatcgcttcttccgtggtcttctgacacctggagagttctttgccaagtgagtttggattttctagctgtttttagg
ctcaatttaaatattaaaattttcagatcgcaaacccggtatcttgggccattcgacgaaatgccaacggaggatacacttcca

gcagccgacgagtacatcattctgcagcgaatgggacgacttccggatagcggtaaatcaatcttcCGTcaAgtcggca
atctgtctacacctaaagatccagccaaacctccggccaaggcacaagttgtgggatggccaccggtgagatcataccgg
aagaacgtgatggtttcctgccaaaaatcaagcggtggcccggaggcggcggcgttcgtgaagGATTACAAGGAT
GACGACGATAAGGATTACAAGGATGACGACGATAAGGAGAACCTCTACTTCCAATCG
ATGAGTAAAGGAGAAGAACTTTTCACTGGAGTTGTCCCAATTCTTGTTGAATTAGATG
GTGATGTTAATGGGCACAAATTTTCTGTCAGTGGAGAGGGTGAAGGTGATGCAACAT
ACGGAAAACTTACCCTTAAATTTATTTGCACTACTGGAAAACTACCTGTTCCATGGgtaa
gtttaaacatatatatactaactaaccctgattatttaaattttcagCCAACACTTGTCACTACTTTCTGTTATG
GTGTTCAATGCTTCTCGAGATACCCAGATCATATGAAACGGCATGACTTTTTCAAGAG
TGCCATGCCCGAAGGTTATGTACAGGAAAGAACTATATTTTTCAAAGATGACGGGAAC
TACAAGACACgtaagtttaaacagttcggtactaactaaccatacatatttaaattttcagGTGCTGAAGTCAA
GTTTGAAGGTGATACCCTTGTTAATAGAATCGAGTTAAAAGGTATTGATTTTAAAGAAG
ATGGAAACATTCTTGGACACAAATTGGAATACAACTTCAACTCACACAATGTATACATC
ATGGCAGACAAACAAAAGAATGGAATCAAAGCTgtaagtttaaacatgattttactaactaactaatctg
atttaaattttcagAACTTCAAAATTAGACACAACATTGAAGATGGAAGCGTTCAACTAGCAG
ACCATTATCAACAAAATACTCCAATTGGCGATGGCCCTGTCCTTTTACCAGACAACCA
TTACCTGACCACACAATCTGCCCTTTCGAAAGATCCCAACGAAAAGAGAGACCACAT
GGTCCTTGTTGAGTTTGTAACAGCTGCTGGGATTACACATGGCATGGATGAACTATAC
AAAtgattcccaggactgaaattgctgcccctcaacgttttatccacatttttccagttgagatcgcatttttatctttaaaaaattt
actaaaccttattgttgccatgagtcataattgtgatatacctataattccttaatataatcaatcttcttgaatccaccccactcatt
gattagttgtgtgcaataataactttcgtttaatgtttgtagtctattggtatcgctgttttagcgtttttatgttgttttataatgaaccaac
accgtttccccatttatacttgcgtaggacaaaaataaatcaattgtctatcaaactcggttcctttcttgtttttttttgaagaattaa
aaaggtatcactcctgtttcgagactatctagtgcgatgtaaatttcgctctcaaaaaacctactaaattaaaaaaagtttgccg
ataaaagtattatttatttttaattattgaatttttaacactctttgcaaagtcttgatgttctgtgaaacttcaaatatttatttaagcgaa
tctgaatgcccttctccaaaaaaattgcatcctatcagtgt

Full-length sequences of *F52F12.8_aid-2×flag-gfp*_donor Red: insertions

ATGGCTGAAAAAAAGCAATACATTTCCACGGAGCCTGCTGATAAGgtatgtctttgcgatcccg
aataataatacaattcatttattgtagATCAAATTTCGCGCGGATCCCAAAGAAGAGCAAAAAACGT
TTCTGAAGATTACCAATAAAAGTGAAATGAAACAGGCATTCAAAGTGAAATGTACTAGA
AACGACTTGTTTCGAATCAAACCGGCTACTGGAATTCTCGATTATAATCAGTCACTCA
CTATTGCTCTTATCTACAGgtttgggagaattttcataaattgtgatttgtctagattgataaacttatatacctaccgg
gatactgtagtggtgggactgtagcggtactgtagggagtactaaaacatctcgattctcagcaacaattttcagccaacctct
tttaaaattgaatgagctttcagAGGCGGTCAAGACAATGTTCCATCAGATGAAAAACATCATTTT
GGTGTCTATCATATTCCTGCACCAGAAGGATGTACTTGTGAAGGTGCTTGGGCTGAA
CACTATGGACCACCACAAGGAGAGCATAAGCTTCGtGTaGTaTGGGAAcctaaagatccagc
caaacctccggccaaggcacaagttgtgggatggccaccggtgagatcataccggaagaacgtgatggtttcctgccaaa
aatcaagcggtggcccggaggcggcggcgttcgtgaagGATTACAAGGATGACGACGATAAGGATTAC
AAGGATGACGACGATAAGGAGAACCTCTACTTCCAATCGATGAGTAAAGGAGAAGAA
CTTTTCACTGGAGTTGTCCCAATTCTTGTTGAATTAGATGGTGATGTTAATGGGCACA
AATTTTCTGTCAGTGGAGAGGGTGAAGGTGATGCAACATACGGAAAACTTACCCTTA
AATTTATTTGCACTACTGGAAAACTACCTGTTCCATGGgtaagtttaaacatatatatactaactaacc
ctgattatttaaattttcagCCAACACTTGTCACTACTTTCTGTTATGGTGTTCAATGCTTCTCGA
GATACCCAGATCATATGAAACGGCATGACTTTTTCAAGAGTGCCATGCCCGAAGGTTA

TGTACAGGAAAGAACTATATTTTTCAAAGATGACGGGAACTACAAGACACgtaagtttaaac
agttcggtactaactaaccatacatatttaaattttcagGTGCTGAAGTCAAGTTTGAAGGTGATACCCTT
GTTAATAGAATCGAGTTAAAAGGTATTGATTTTAAAGAAGATGGAAACATTCTTGGACA
CAAATTGGAATACAACTTCAACTCACACAATGTATACATCATGGCAGACAAACAAAAG
AATGGAATCAAAGCTgtaagtttaaacatgattttactaactaactaatctgatttaaattttcagAACTTCAAAAT
TAGACACAACATTGAAGATGGAAGCGTTCAACTAGCAGACCATTATCAACAAAATACT
CCAATTGGCGATGGCCCTGTCCTTTTACCAGACAACCATTACCTGACCACACAATCT
GCCCTTTCGAAAGATCCCAACGAAAAGAGAGACCACATGGTCCTTGTTGAGTTTGTA
ACAGCTGCTGGGATTACACATGGCATGGATGAACTATACAAATGAacttctgctcttgaaataaa
ttatttcattgattttattagattttacgaaaaatttttggcaaaccctcatttactagcgtgctcatgtacttgaacgctcaatcccta
acgatggctcaatcccttcgacgctcatttttaccaagaaactgtagctaaaacacaattcagaatacattatcactttcaaaa
aaaaattctgcaaacataatttcttcaaagtttcaaaaaagttccagattatttttggaaaagtttggaatagcattcaatgtgttta
atttaatgtaactacacccgcgacaatttcctaaccaactccaaccaaatcttcaaacgcttaaatcctcttcaaaagcttaagt
tcaaattttccaacactacagtaatcatacagtaagcctac

Full-length sequences of *ZK596.2_aid-2×flag-gfp*_donor   Red: insertions

TTGAGGATTTGCACAACATTGGATTCTTGCACCGAGACATTAAACCAGGAAATTATAC
CATCGGACGGAAGGAGATGCACGAATTGAGAAAGgttgttaaataattttacgtatttcaaaaacgtttc
gtacttcagGTCTACATGCTCGATTTCGGAATGGCACGCAAGTTTGCCCGTGAAGACGGA
ACTCTTCGCAATCCACGTGCTCGTGCTGGATTCCGTGGAACGGTGAAGTATGCTCCA
TTGGCTTGCCATATTCAACGTGAGCAGTGTCGCAAGGATGACATTGAAAGTTGGCTC
TACATGGTCGTTGAAATGACATGTGGACGTCTTCCCTGGCGTAATCTGACTGAAAGT
GATGATGTGGGAGTGTTCAAGAAGGAATGTAAAACGACTCGTCTTAGGTGTCTGTTT
GGAGGTTGTCCGCGTGAATTCACTGAAGTTTTCCCGATTCTTGACAAAGGAAAGTTC
TTCGATGCTCCGGAGTACACGACCATCTACGAGCTCCTTGAAAAGGCAATGGTTAAC
ACCAAATCGAACGAGTTTCCaTAcGAcTGGGAGcctaaagatccagccaaacctccggccaaggca
caagttgtgggatggccaccggtgagatcataccggaagaacgtgatggtttcctgccaaaaatcaagcggtggcccgga
ggcggcggcgttcgtgaagGATTACAAGGATGACGACGATAAGGATTACAAGGATGACGACG
ATAAGGAGAACCTCTACTTCCAATCGATGAGTAAAGGAGAAGAACTTTTCACTGGAGT
TGTCCCAATTCTTGTTGAATTAGATGGTGATGTTAATGGGCACAAATTTTCTGTCAGTG
GAGAGGGTGAAGGTGATGCAACATACGGAAAACTTACCCTTAAATTTATTTGCACTAC
TGGAAAACTACCTGTTCCATGGgtaagtttaaacatatatatactaactaaccctgattatttaaattttcagCCA
ACACTTGTCACTACTTTCTGTTATGGTGTTCAATGCTTCTCGAGATACCCAGATCATAT
GAAACGGCATGACTTTTTCAAGAGTGCCATGCCCGAAGGTTATGTACAGGAAAGAAC
TATATTTTTCAAAGATGACGGGAACTACAAGACACgtaagtttaaacagttcggtactaactaaccata
catatttaaattttcagGTGCTGAAGTCAAGTTTGAAGGTGATACCCTTGTTAATAGAATCGAG
TTAAAAGGTATTGATTTTAAAGAAGATGGAAACATTCTTGGACACAAATTGGAATACAA
CTTCAACTCACACAATGTATACATCATGGCAGACAAACAAAAGAATGGAATCAAAGCT
gtaagtttaaacatgattttactaactaactaatctgatttaaattttcagAACTTCAAAATTAGACACAACATTGA
AGATGGAAGCGTTCAACTAGCAGACCATTATCAACAAAATACTCCAATTGGCGATGGC
CCTGTCCTTTTACCAGACAACCATTACCTGACCACACAATCTGCCCTTTCGAAAGATC
CCAACGAAAAGAGAGACCACATGGTCCTTGTTGAGTTTGTAACAGCTGCTGGGATTA
CACATGGCATGGATGAACTATACAAATAGtcaataaatattttgaattattcatctttatcttcttttagtggaatat
gttttacataaaagtttcacaaaatttcagtttacaatgaatcatgacttcgcgtgcgattcgccgcaacattttcgaattggacat

atgtggatgttgaaataaaattaagagtaaaaccaacttgagtatccccataaatttttttatcaatagaaccgataattattttg aacaagcaaaacaaatgaggatatgaaagttaaaaacaatcgtttagttaaaggatcaataataaaaaactaaatgaaa catctcagatatacagataataatggtggctacattgtagtgtgc

Full-length sequences of him-8_mutant_donor Blue: mutations
tcgagcaattgcttggaaaagaaagtacagaaagctccgtcttatcgatcacgcattg

Supplementary Table S10. List of primers used for qPCR experiments, Related to methods

Gene	Forward (5' → 3')	Reverse (5' → 3')
F22D6.9	*gctcgtatgaaacctggcactc*	*tggccatccattcatatccatcatc*
F38H4.4	*gaggatgccaacattgcagc*	*cggcaatggcagttgagttag*
gsp-3	*gtctttggacaggatgttgttgc*	*acatgacgaaagtgcagacc*
gsp-4	*tgacatcgacttggttgctcg*	*ctatcctttacgaagagacttcggag*
egg-4	*aggaacgtcacgtggaacatt*	*tggacagctcgttgtactgg*
F46F11.1	*cgatgtttggagaataaagagtctg*	*aaattcatagcacgttgccac*
F54C8.4	*aagaataagagaaaacaccgtgaaatt*	*tgcaacaggttgattgagttg*
scpl-4	*aagaaatactcgcattggagca*	*ctggcaatggatcaggcaaca*
K09F6.3	*actcacacagtgaagcacta*	*agattcacggtggaagttgtg*
Y48G1C.5	*gcccgagaaactactgatcac*	*gttagagtagatttggcgcattg*
Y71G12B.30	*aatcatgagagtcgaccggtgaa*	*gacgtcggcaatgtcagttgga*
ZK354.8	*aagtgagcaaattgaataagcgt*	*ttcatcgcgacttctggctc*
W03F11.4	*aacactgcctccaccacc*	*gttcctttctcatgcacttcaaa*
alg-3	*ggataacaacacgctgactagttcag*	*cctcgcgcatgttgagacag*
alg-4	*ggataacaacacgctgactagttcag*	*acgcatattgagacaagccaactc*

Supplementary Fig. S1

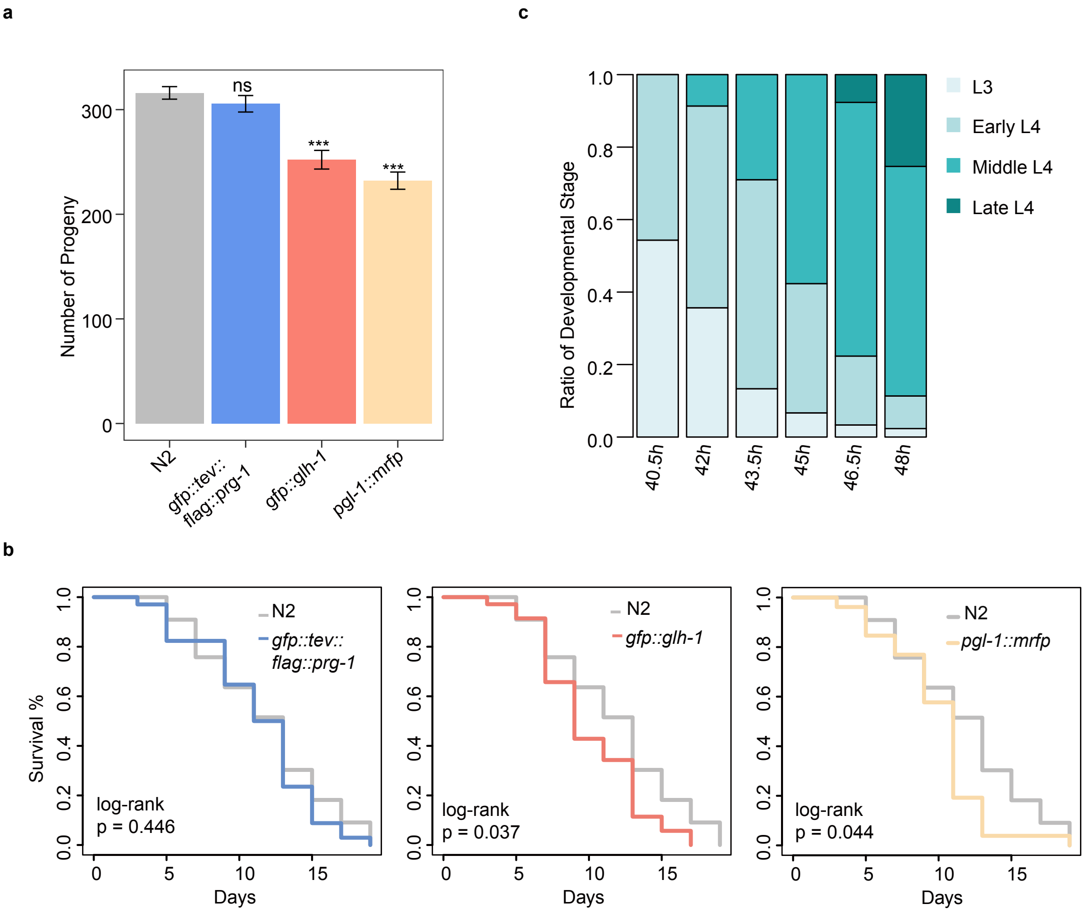

Supplementary Fig. S2

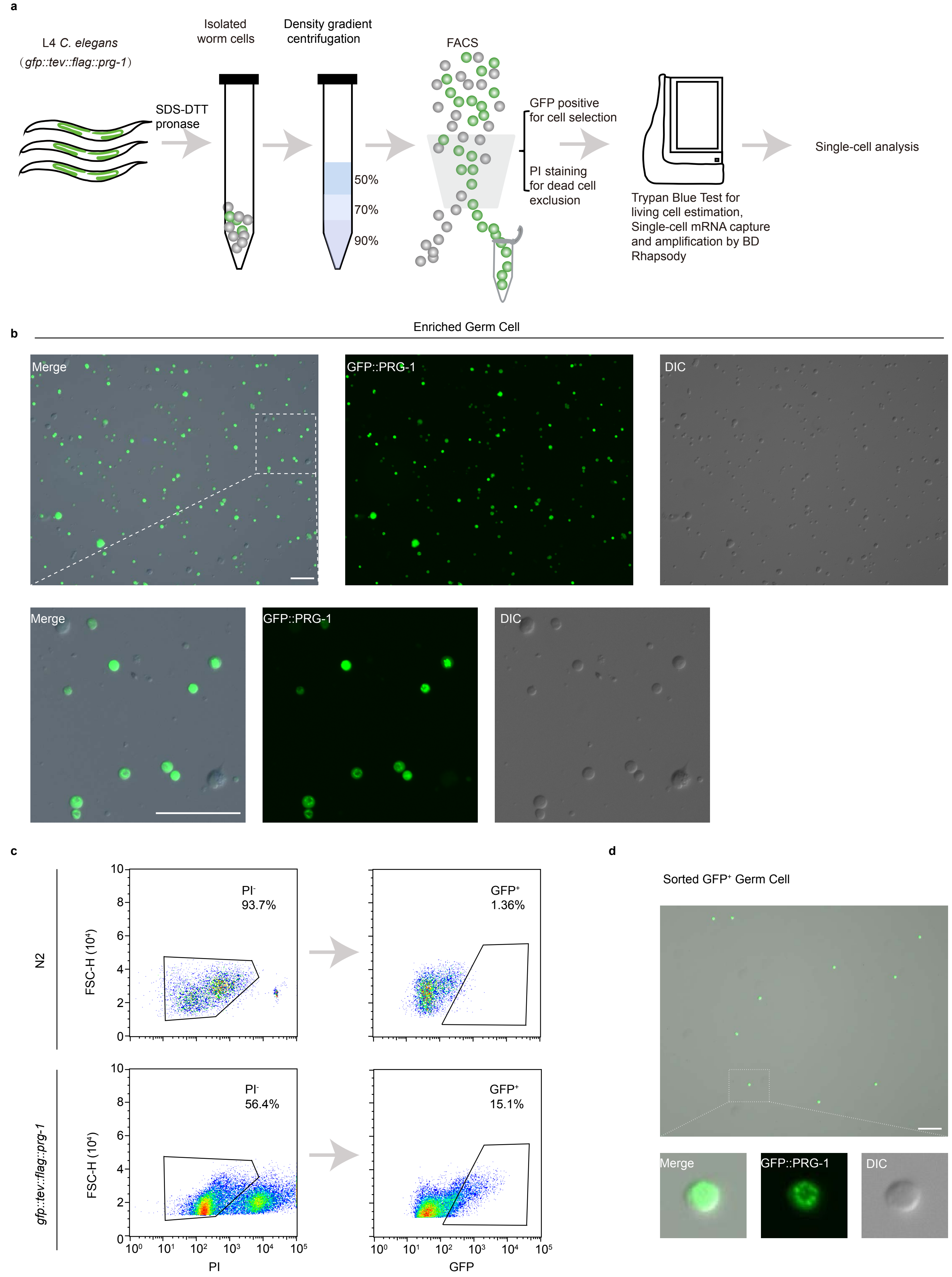

Supplementary Fig. S3

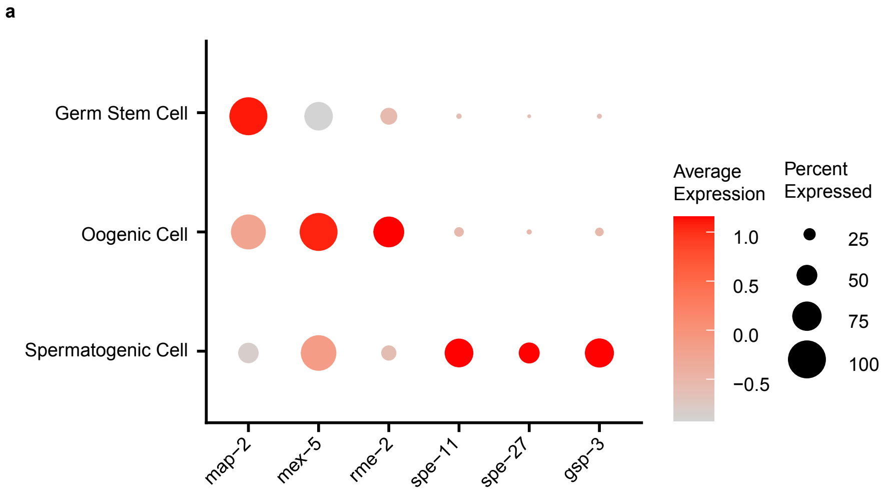

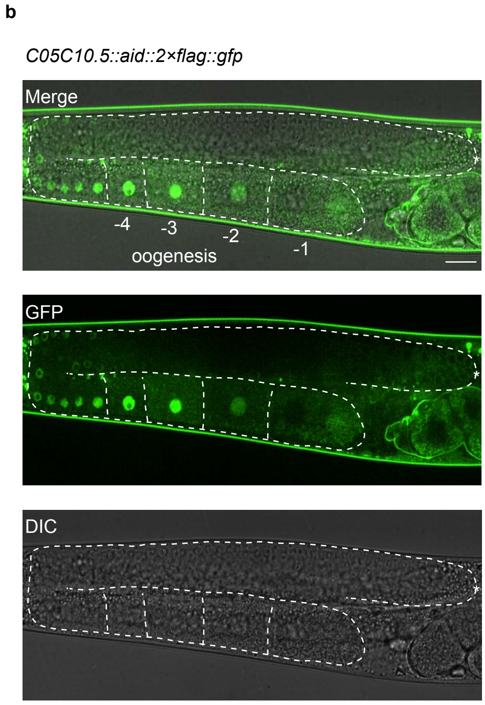

Supplementary Fig. S4

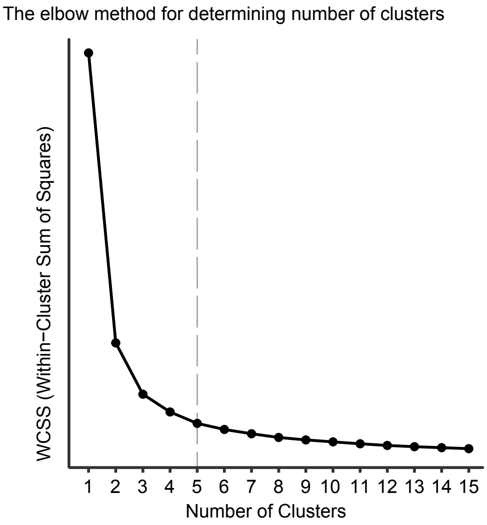

Supplementary Fig. S5

a

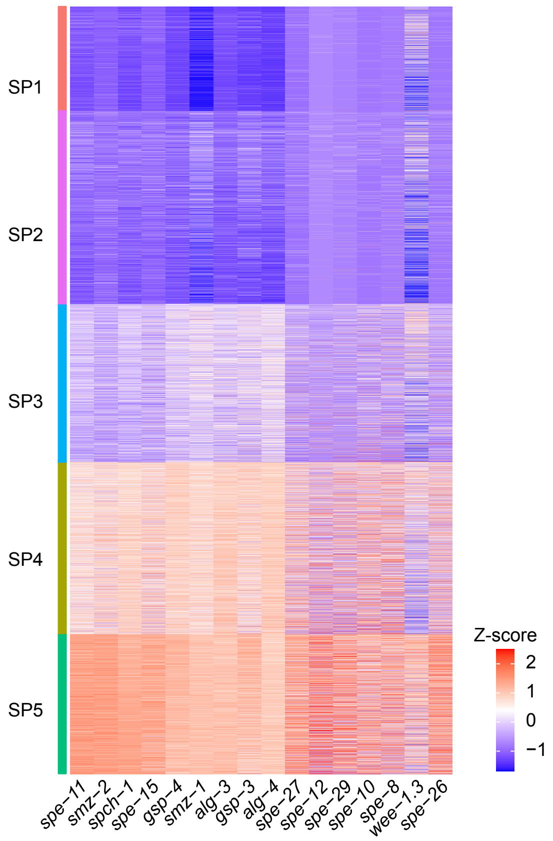

b

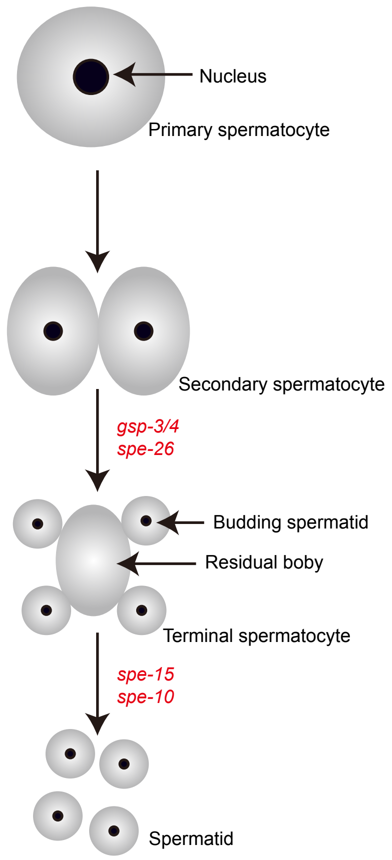

Supplementary Fig. S6

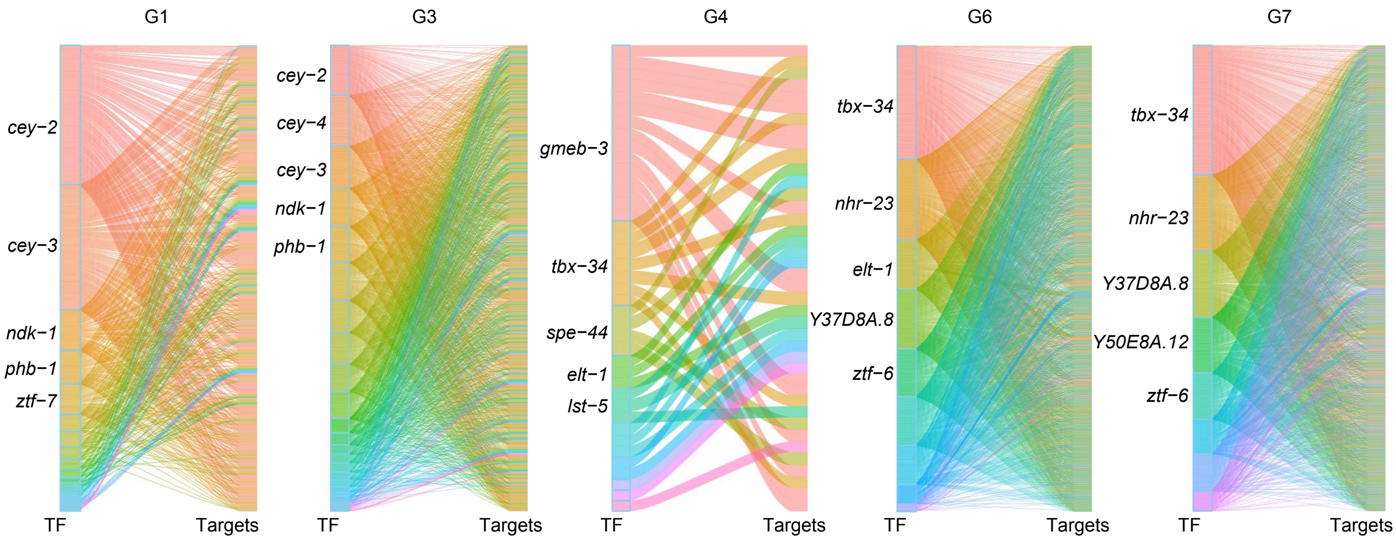

Supplementary Fig. S7

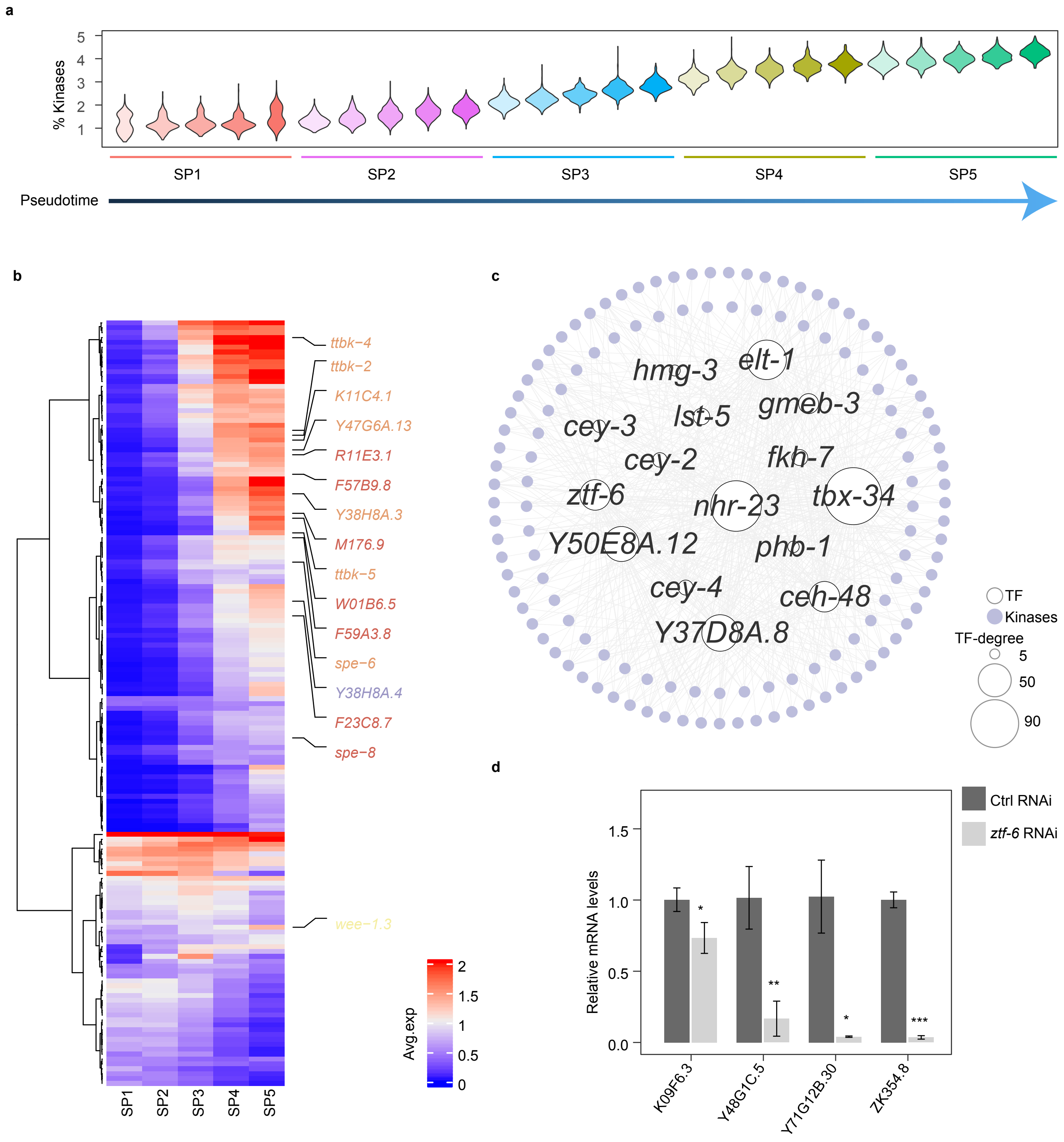

Supplementary Fig. S8

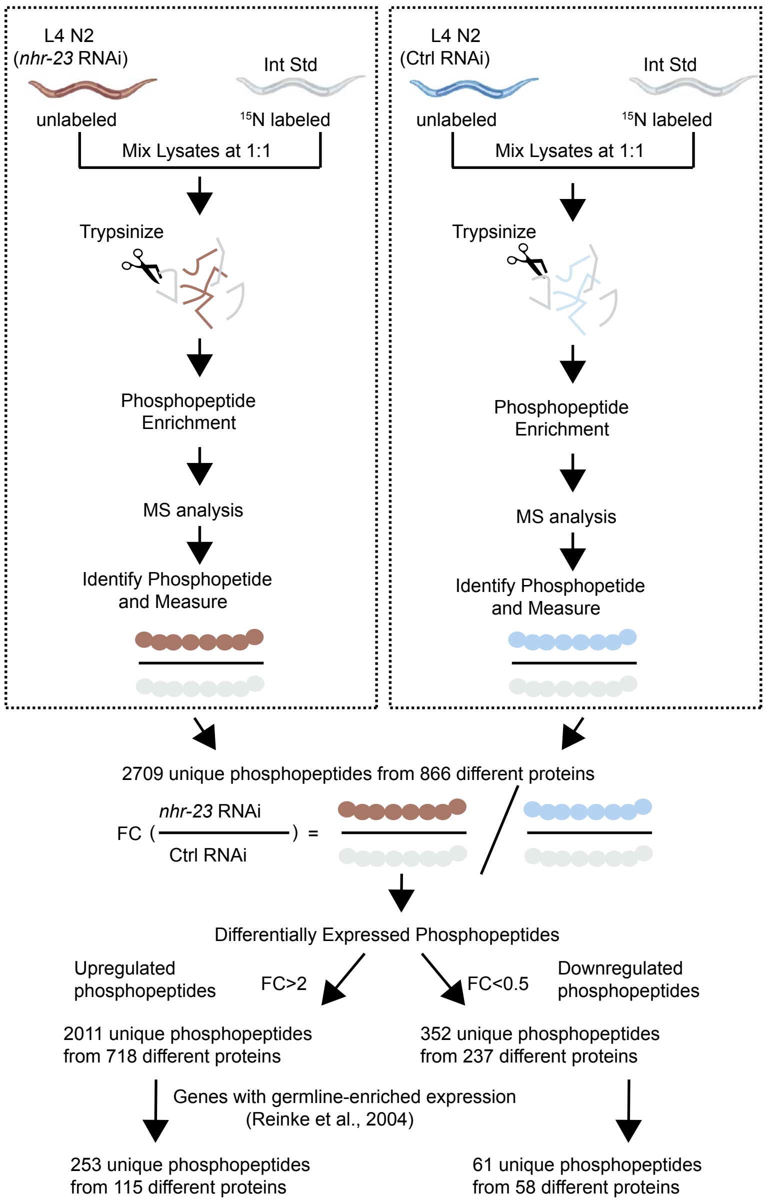

Supplementary Fig. S9

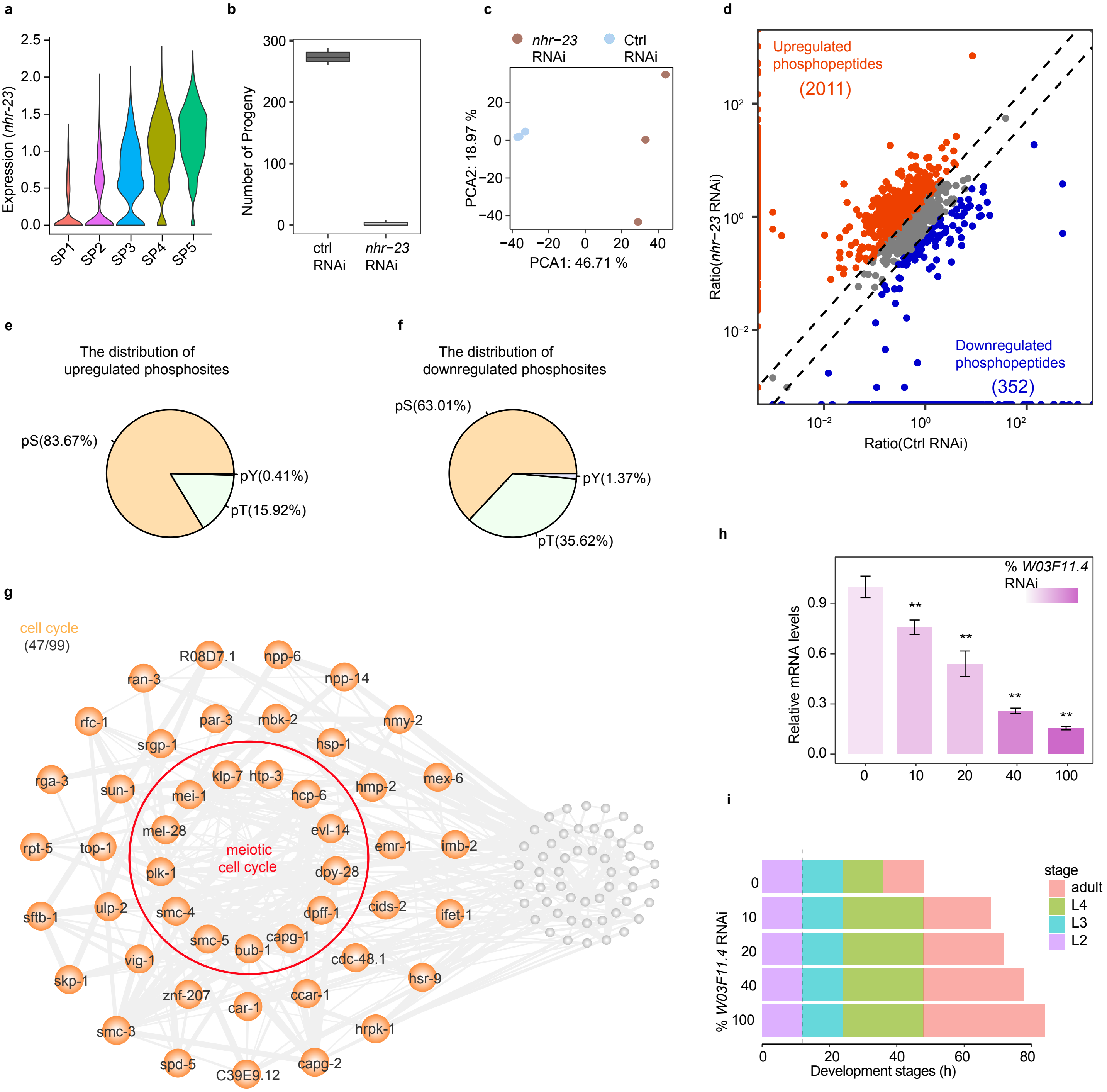

Supplementary Fig. S10

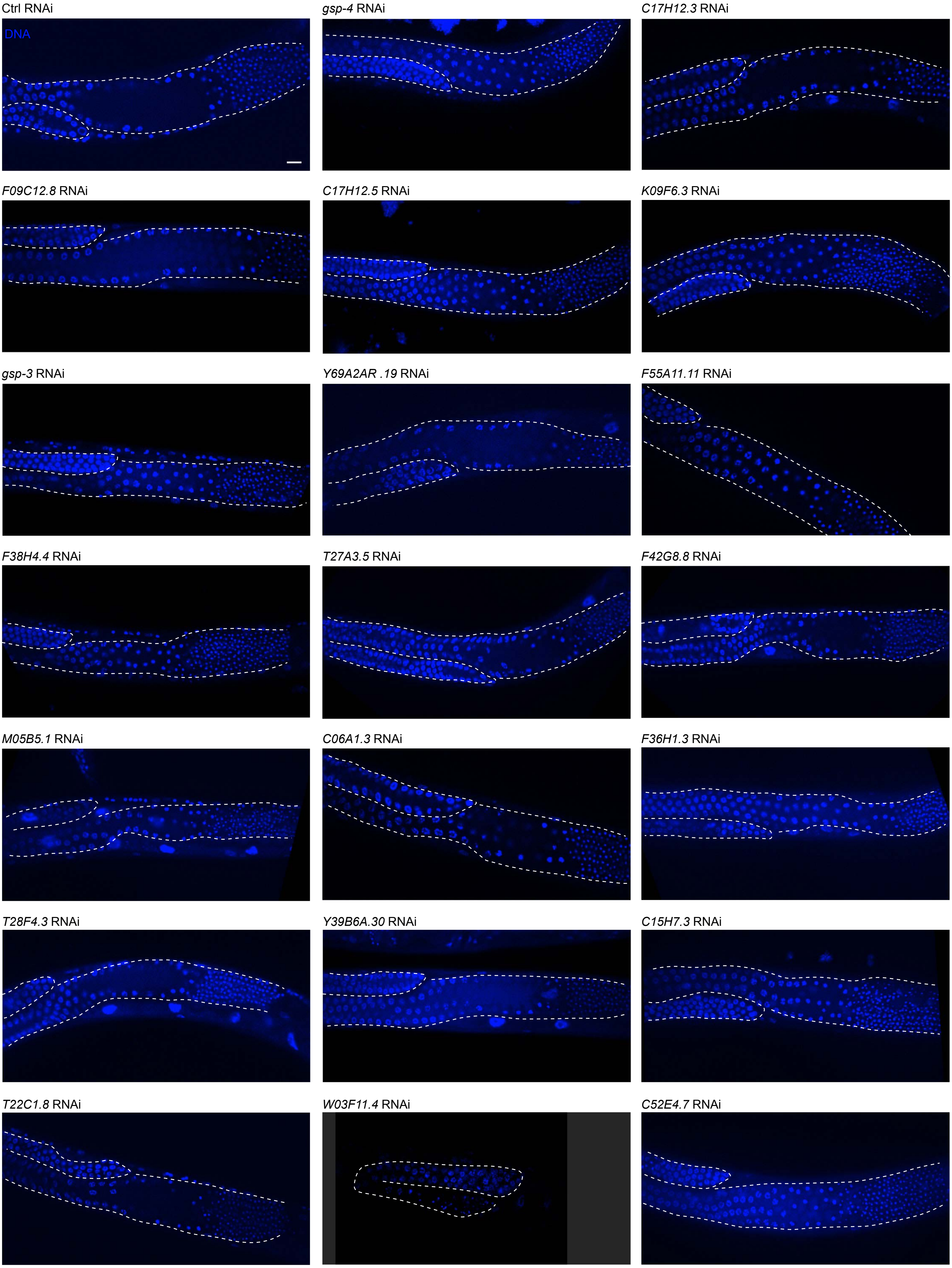

Supplementary Fig. S11

a

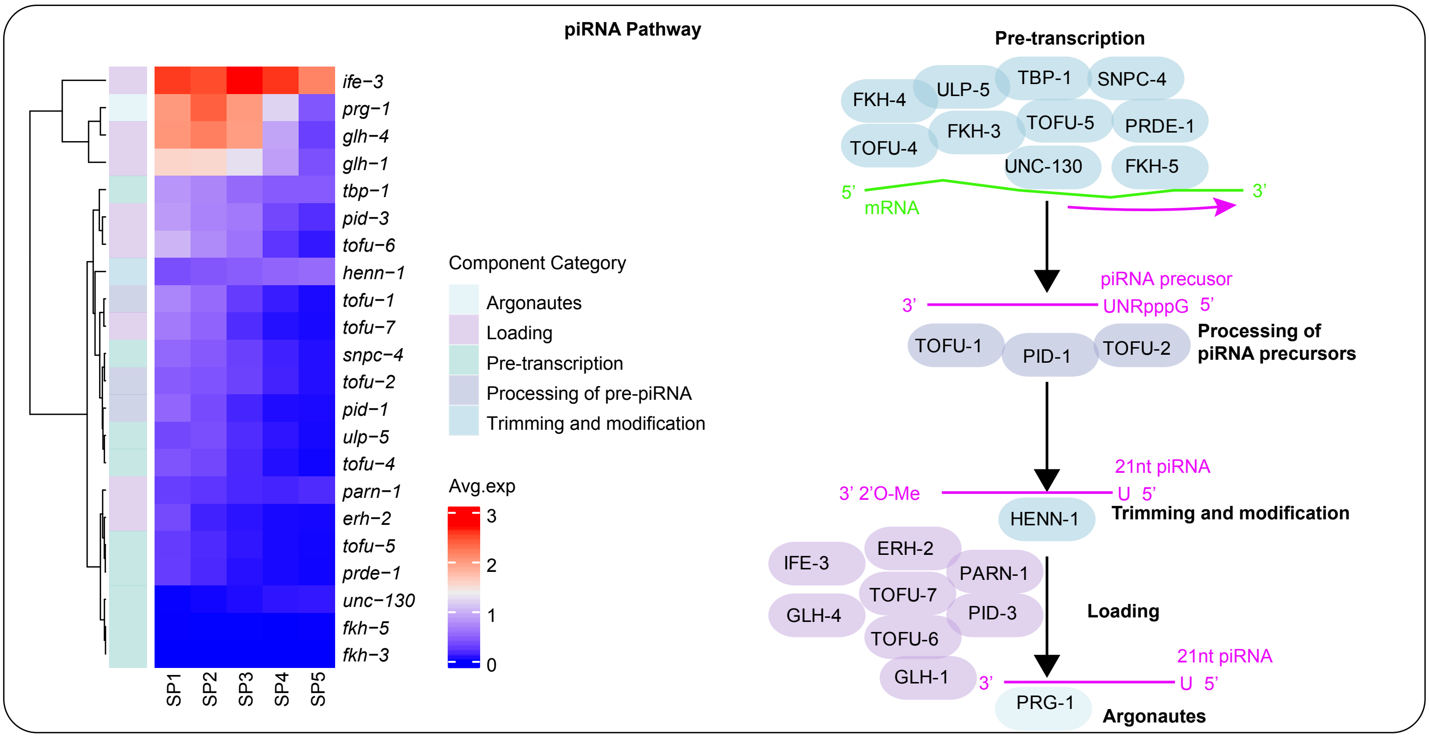

b

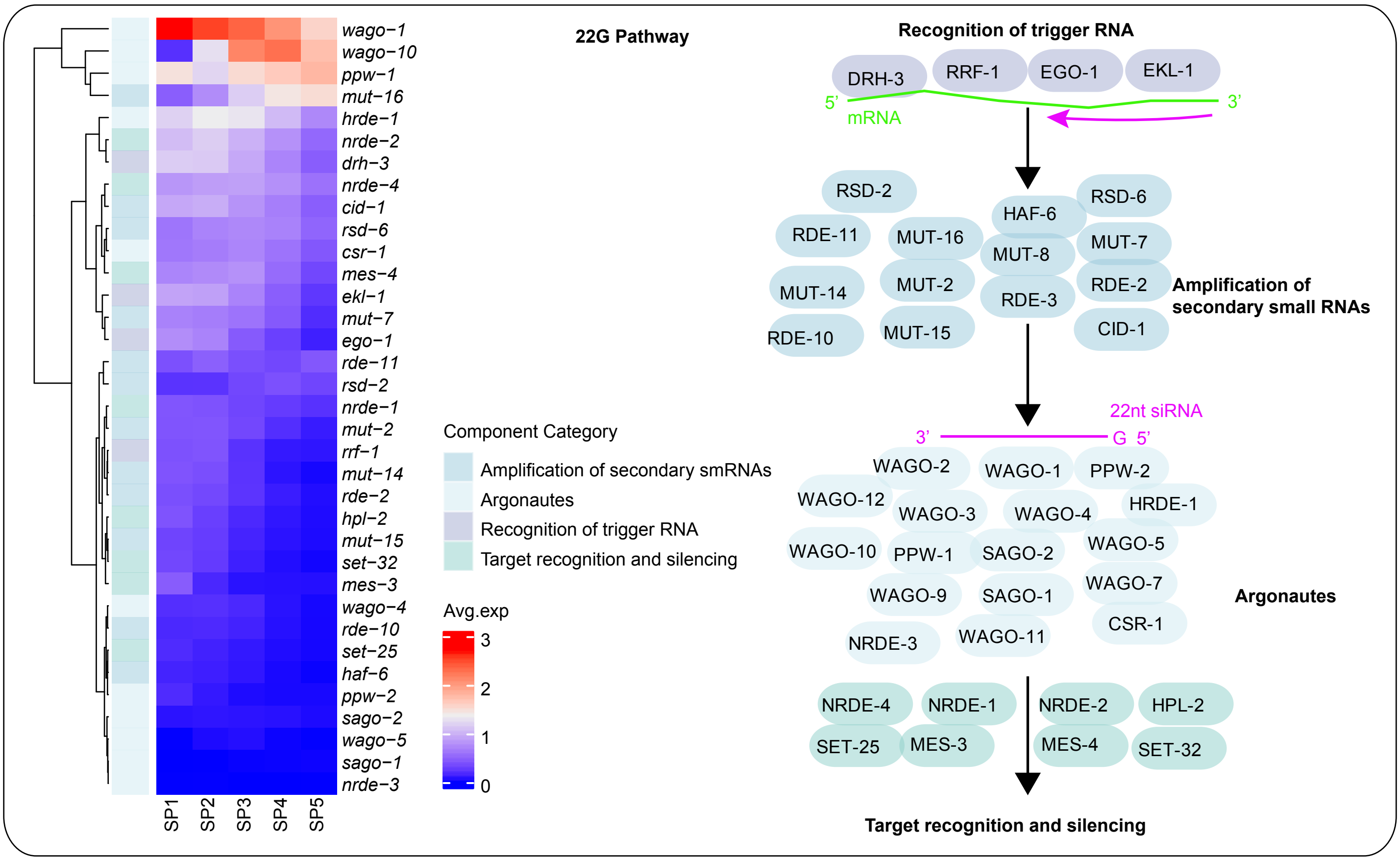

Supplementary Fig. S11

c

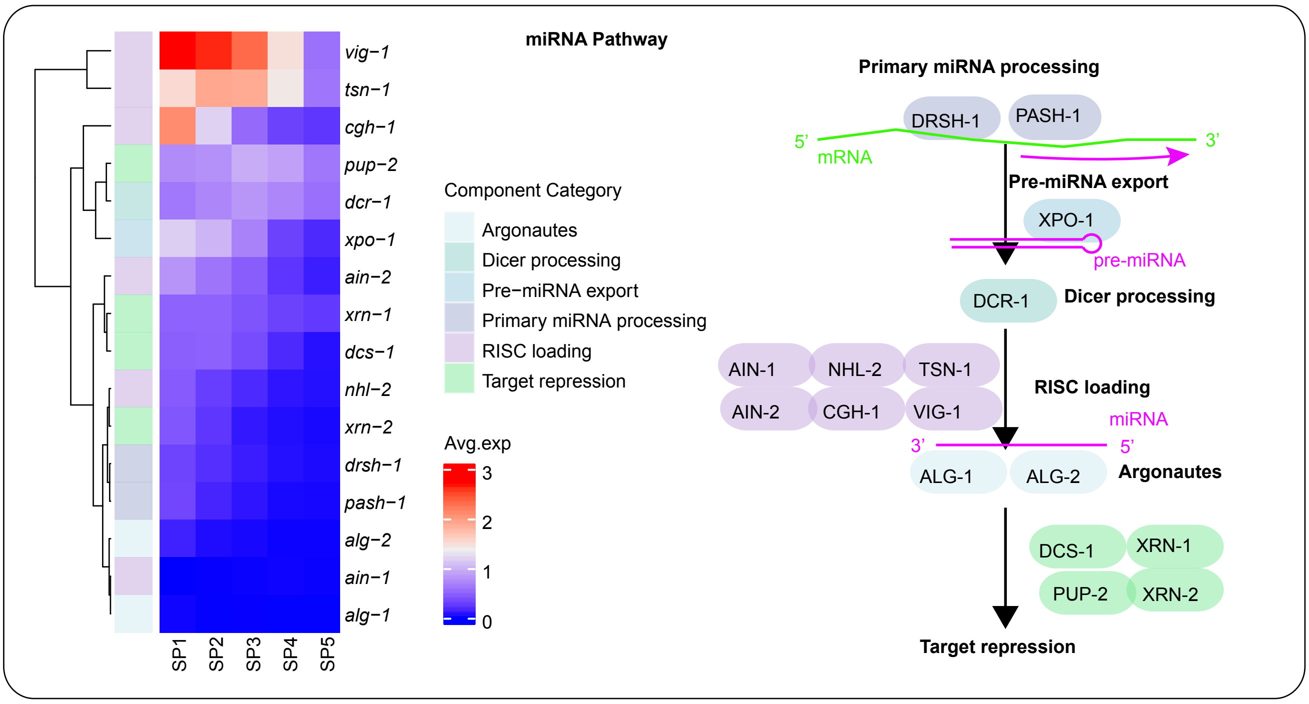

d

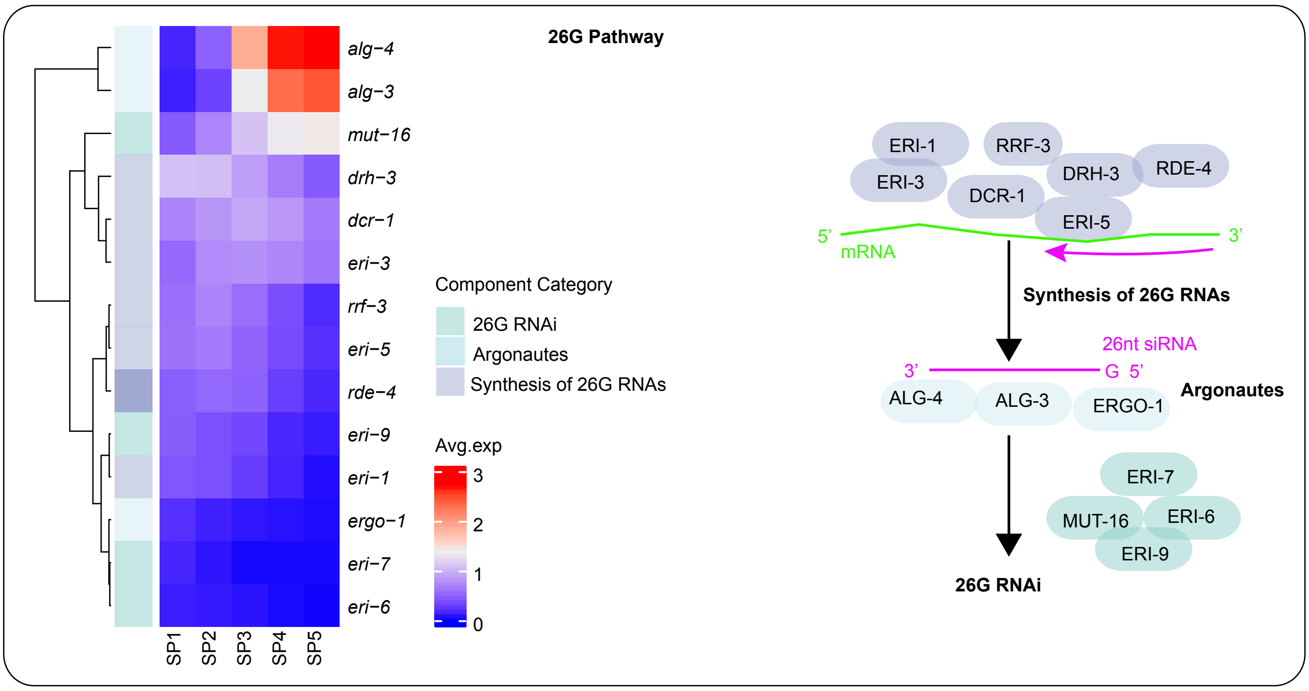

e

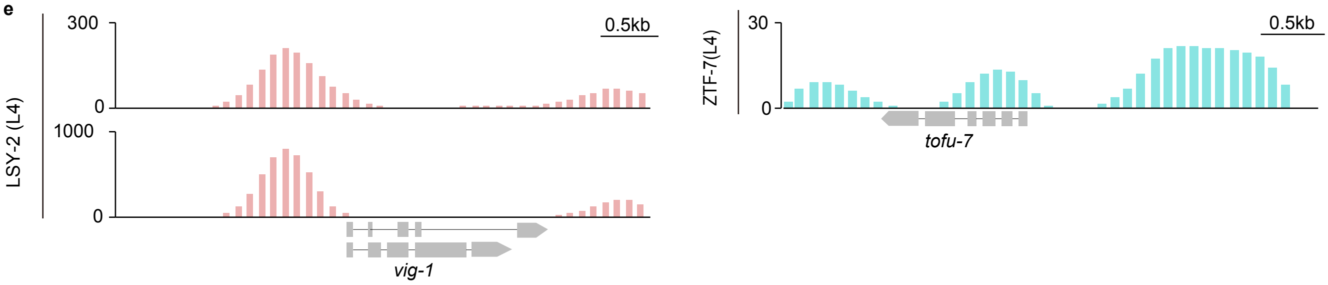

Supplementary Fig. S12

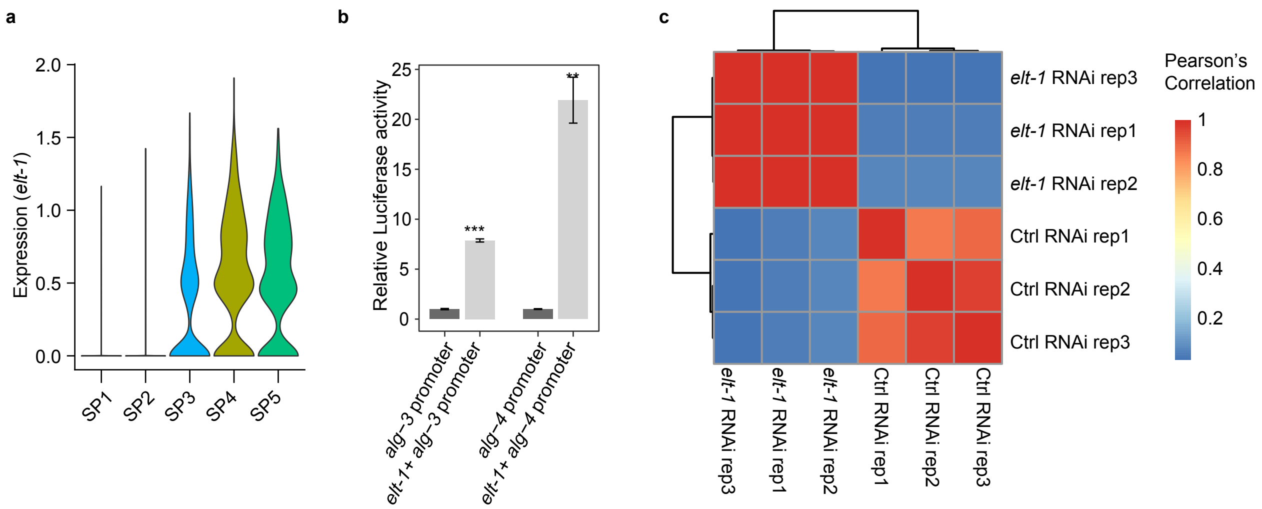

Supplementary Fig. S13

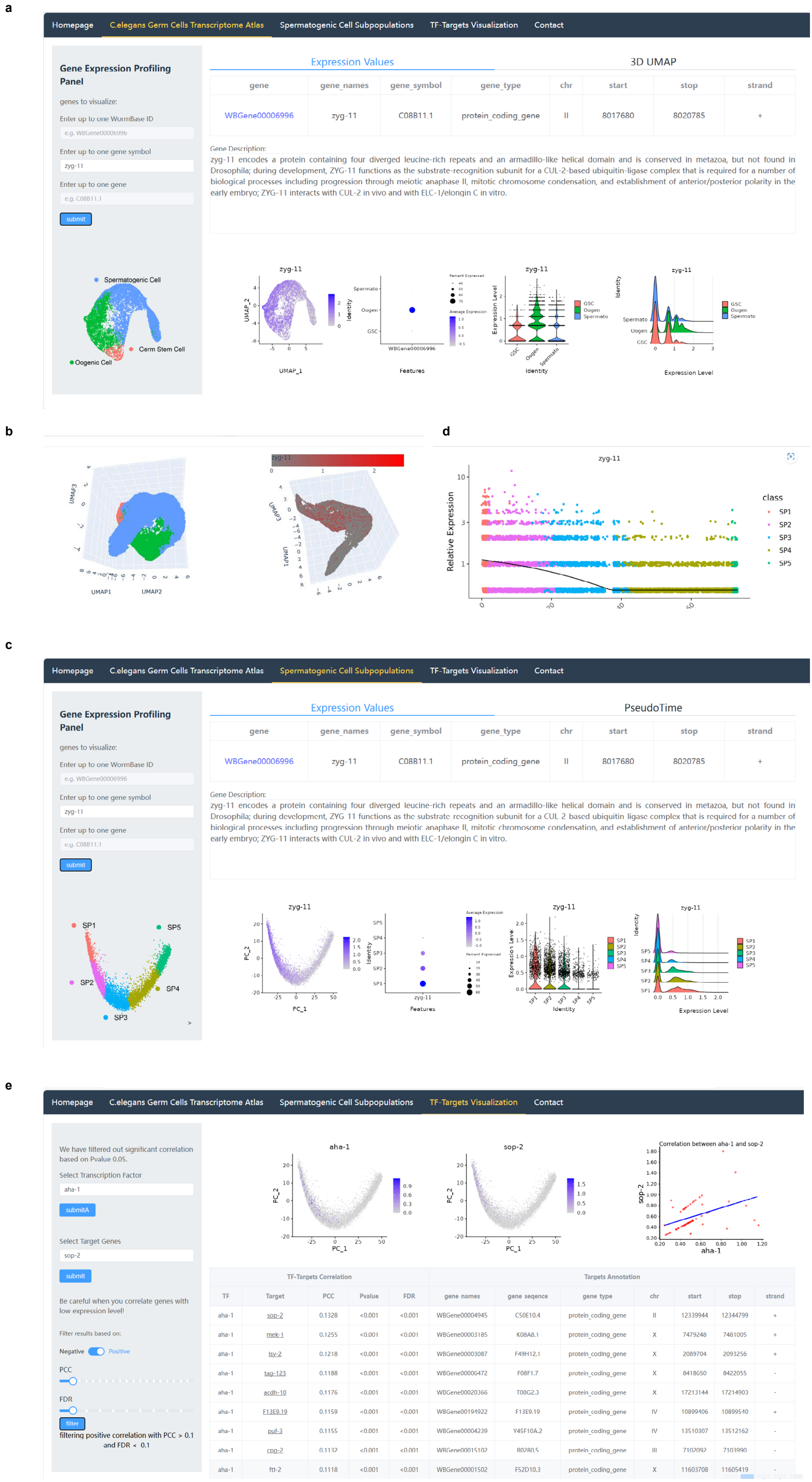

Supplementary Fig. S14

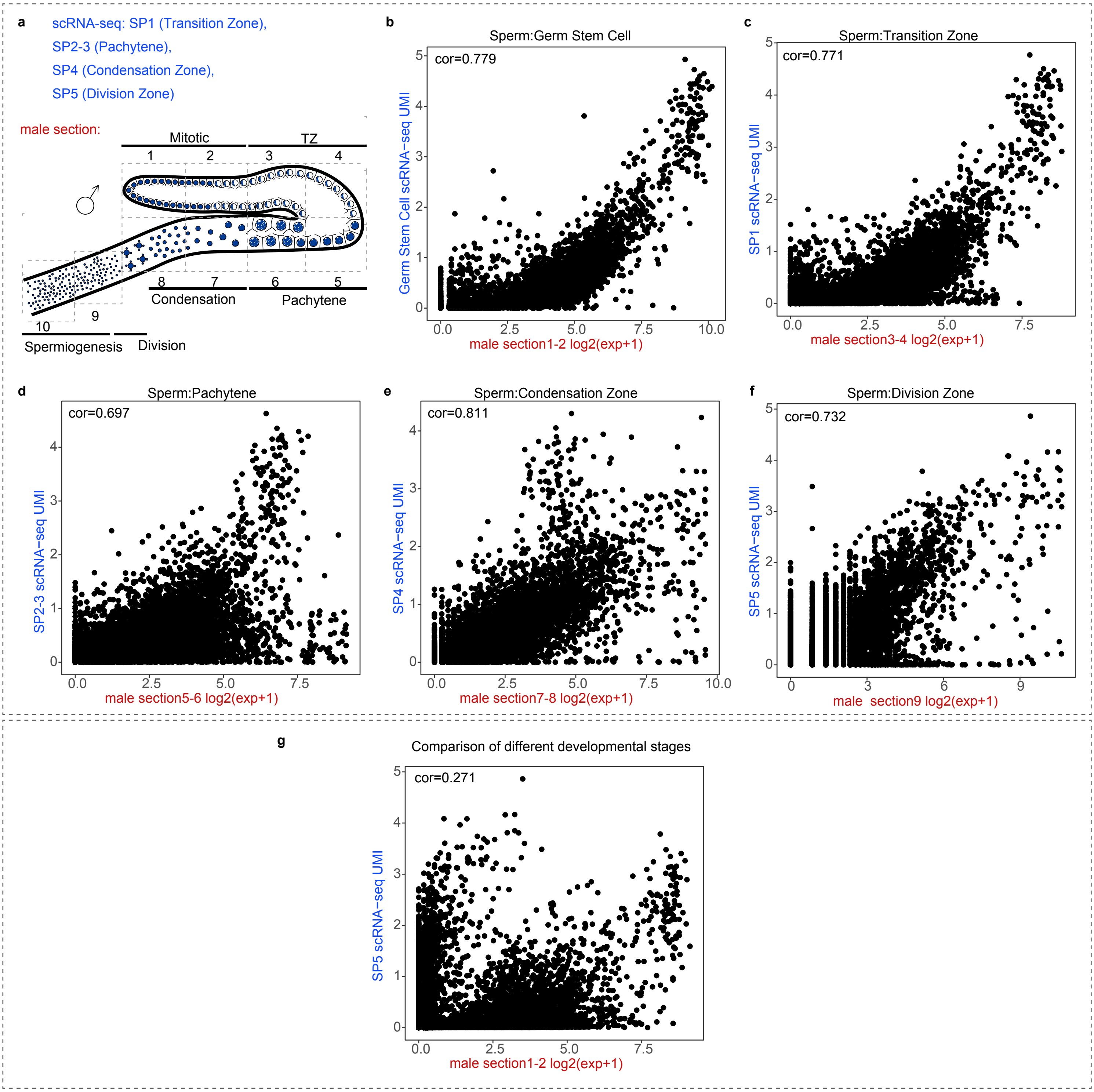

Supplementary Fig. S15

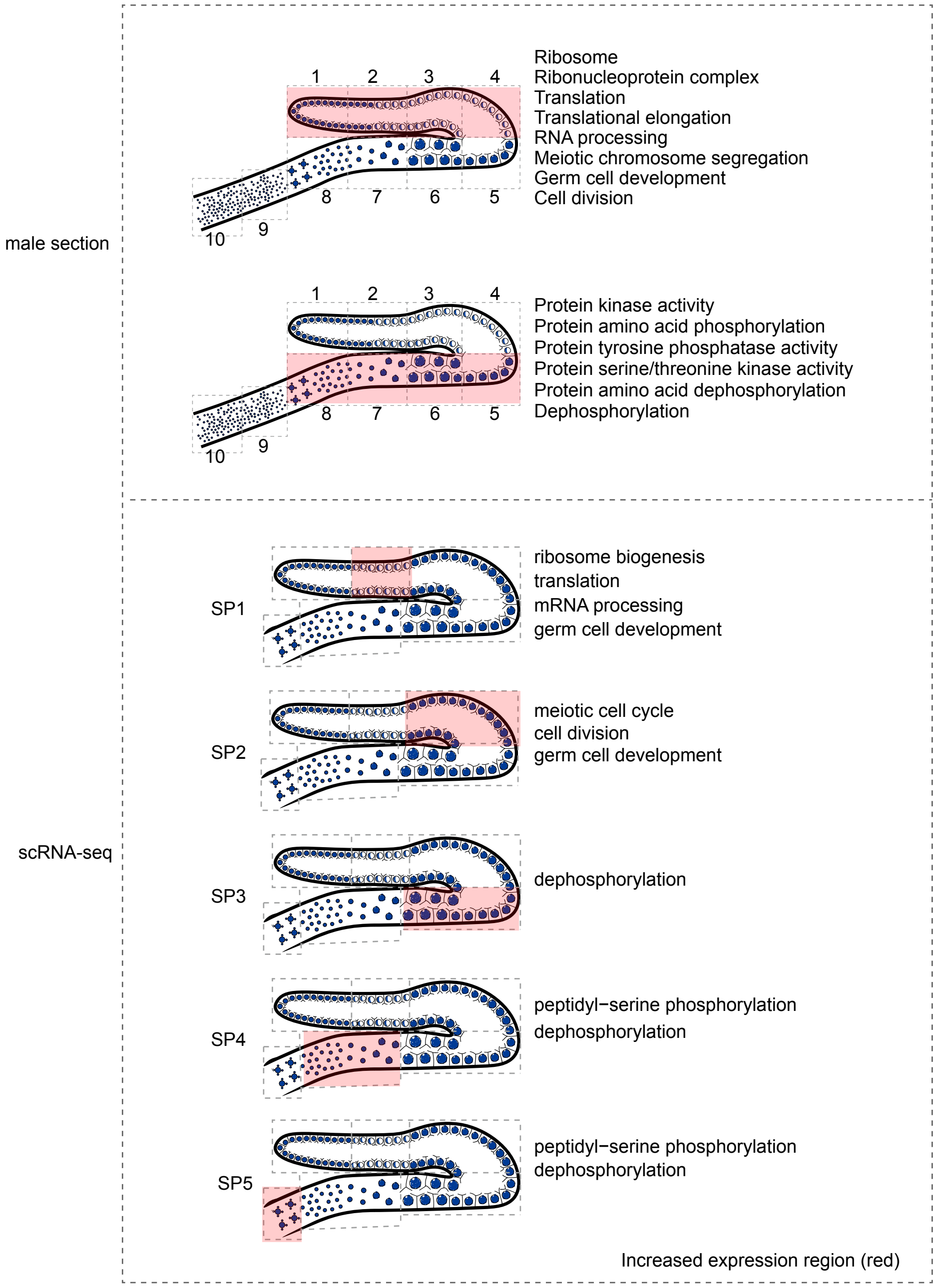

Supplementary Fig. S16

a

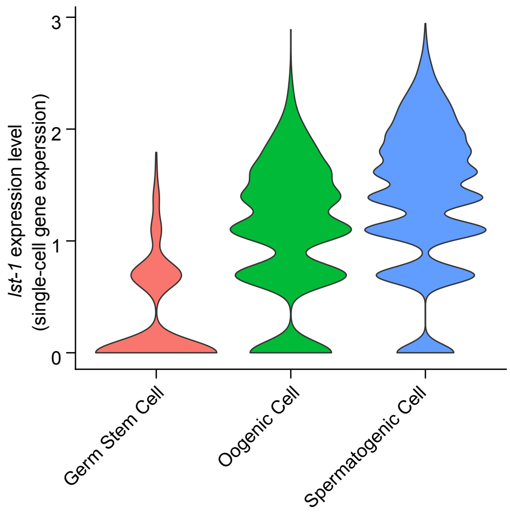

b

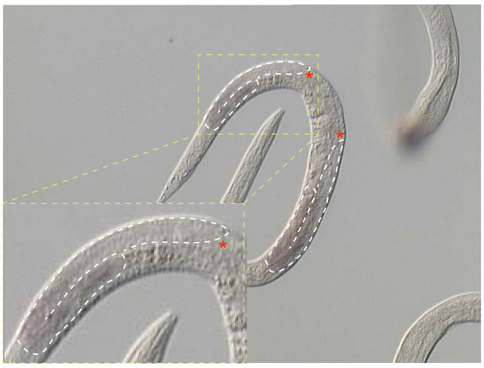

c

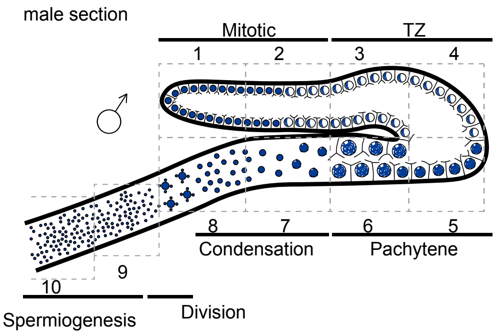

d

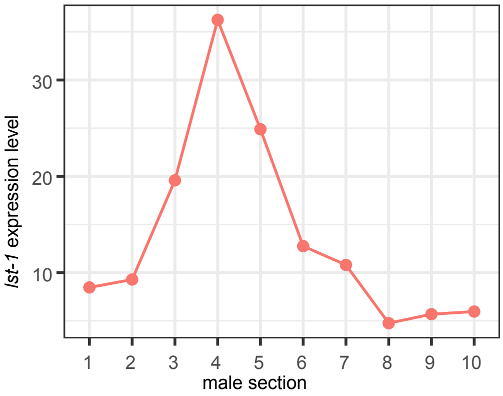

Supplementary Fig. S17

a

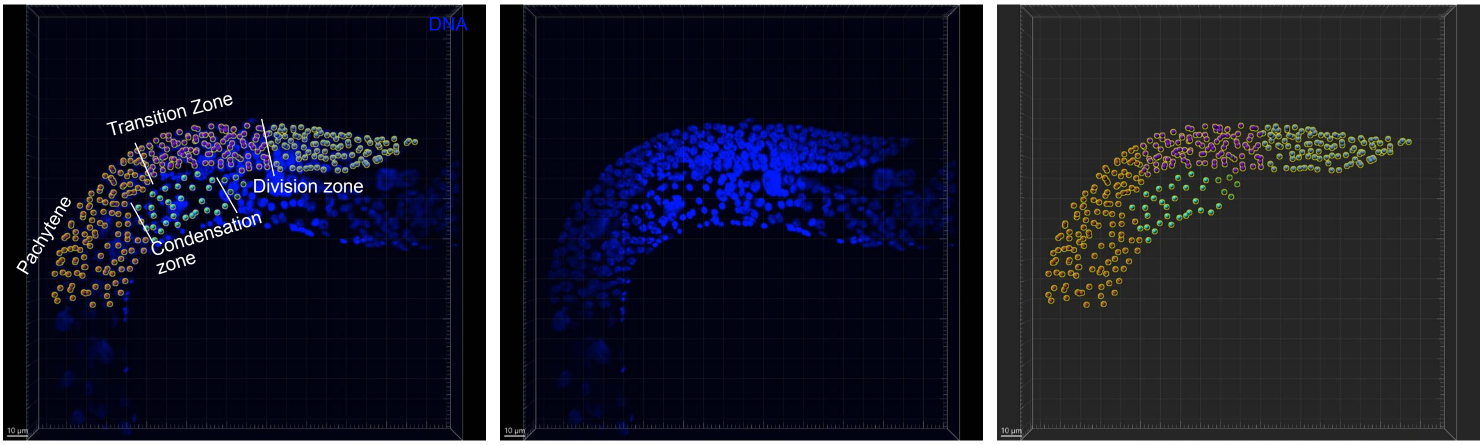

b

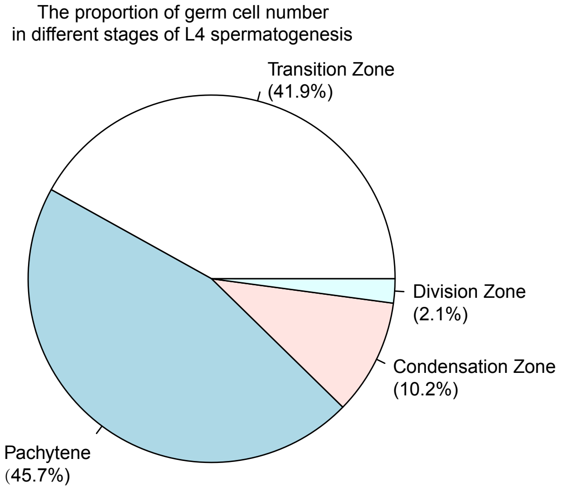

c

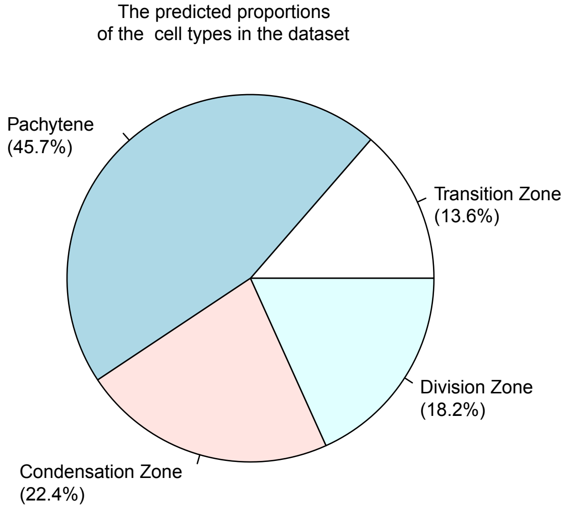

Supplementary Fig. S18

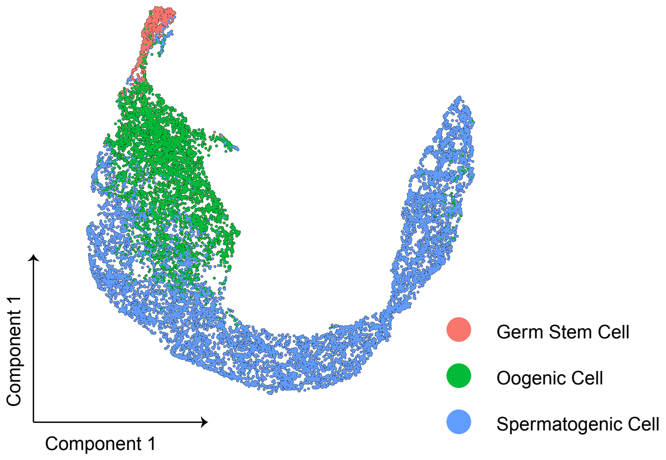

Supplement: Supplementary file 1 — Supplementary Information [file 41421_2025_790_MOESM1_ESM.pdf]
